# Global biogeographic patterns in bipolar moss species

**DOI:** 10.1098/rsos.170147

**Published:** 2017-07-12

**Authors:** E. M. Biersma, J. A. Jackson, J. Hyvönen, S. Koskinen, K. Linse, H. Griffiths, P. Convey

**Affiliations:** 1 Department of Plant Sciences, University of Cambridge, Downing Street, Cambridge CB2 3EA, UK; 2 British Antarctic Survey, Natural Environment Research Council, High Cross, Madingley Road, Cambridge CB3 0ET, UK; 3 Finnish Museum of Natural History (Botany) and Viikki Plant Science Centre, Department of Biosciences, University of Helsinki, PO Box 7, Helsinki FIN-00014, Finland; 4 Department of Biochemistry, University of Turku, Turku, 20014, Finland; 5 National Antarctic Research Center, Institute of Graduate Studies, University of Malaya, 50603 Kuala Lumpur, Malaysia

**Keywords:** bipolar disjunction, bryophyte, Polytrichaceae, *Polytrichastrum*

## Abstract

A bipolar disjunction is an extreme, yet common, biogeographic pattern in non-vascular plants, yet its underlying mechanisms (vicariance or long-distance dispersal), origin and timing remain poorly understood. Here, combining a large-scale population dataset and multiple dating analyses, we examine the biogeography of four bipolar Polytrichales mosses, common to the Holarctic (temperate and polar Northern Hemisphere regions) and the Antarctic region (Antarctic, sub-Antarctic, southern South America) and other Southern Hemisphere (SH) regions. Our data reveal contrasting patterns, for three species were of Holarctic origin, with subsequent dispersal to the SH, while one, currently a particularly common species in the Holarctic (*Polytrichum juniperinum*), diversified in the Antarctic region and *from* here colonized both the Holarctic and other SH regions. Our findings suggest long-distance dispersal as the driver of bipolar disjunctions. We find such inter-hemispheric dispersals are rare, occurring on multi-million-year timescales. High-altitude tropical populations did not act as trans-equatorial ‘stepping-stones’, but rather were derived from later dispersal events. All arrivals to the Antarctic region occurred well before the Last Glacial Maximum and previous glaciations, suggesting that, despite the harsh climate during these past glacial maxima, plants have had a much longer presence in this southern region than previously thought.

## 1. Introduction

Since the nineteenth century, scientists have been puzzled by the origin and evolution of plants with disjunct distributions, and particularly with the most extreme pattern of all—bipolar disjunctions [1–3]. Bipolar distributions characterize species occupying high-latitudinal areas of both the Northern (NH) and Southern Hemispheres (SH), with or without small intermediate populations at higher elevations in the tropics [4]. The distribution pattern could originate from: (i) long-distance dispersal, either in one event or gradually, via high-altitude intermediate latitude ‘stepping-stone’ populations, or (ii) vicariance, with a large ancestral distribution split into smaller units by environmental barriers such as past climate change (e.g. glaciations, sea-level change) or tectonic events.

As bipolar disjunctions in mosses are common (e.g. approx. 45% of all mosses currently occurring in the Antarctic are bipolar [5]), they have received much attention in descriptive studies [4–8]. The disjunction has been suggested to be of post-Pleistocene Holarctic origin, resulting from dispersal along tropical mountain chains across the tropics [5], from where the taxa were able to colonize many high-latitude SH areas left barren by receding glaciers. However, few molecular studies have addressed the question to date. Two recent molecular phylogeographical studies of bryophytes with disjunct distributions reaching as far south as Tierra del Fuego have suggested the distribution to be due to dispersal events, either recent (e.g. *Cinclidium stygium* Sw., no molecular dating but very low variation between hemispheres [9]) or in the more distant past (e.g. the dung-moss genus *Tetraplodon* Bruch & Schimp. dispersed to South America approx. 8.6 Ma [10]). However, the lack of variation in disjunct populations of *C. stygium* makes it difficult to distinguish whether the disjunction is natural or caused by anthropogenic vectors [9], and the dung-associated lifestyle of *Tetraplodon* makes this moss a likely candidate for adventitious dispersal via migrating birds [10] (e.g. becoming attached when birds forage for insects attracted to dung), which might not be a typical characteristic of the majority of bipolar moss species. In-depth investigations into global patterns of dispersal of bipolar mosses are clearly needed, including species that are more widespread and have a more typical bryophyte-representative ecology (i.e. unlike the dung-associated *Tetraplodon*, see above).

**Figure 1.**
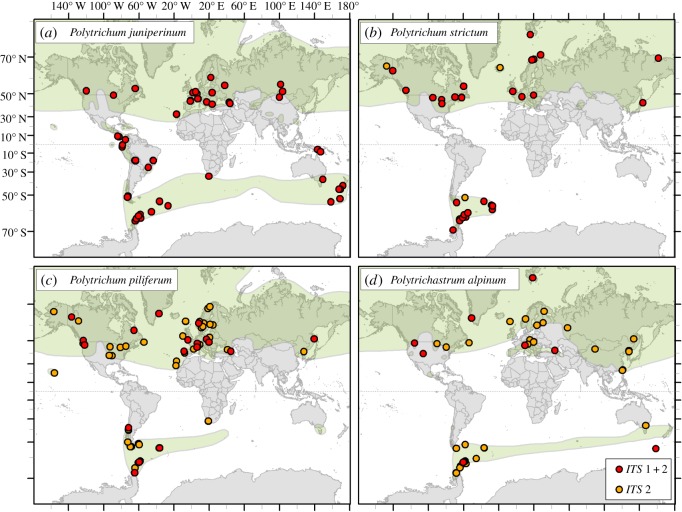
Locations of *ITS* 1 + 2 (red) and *ITS* 2 only (orange) samples of *P. juniperinum* (*a*), *P. strictum* (*b*), *P. piliferum* (*c*) and *P. alpinum* (*d*). Known global distributions of the different species (shown in green) are reproduced from [5].

We here obtained the first large-scale global population dataset (*n* = 255) to explicitly explore the biogeographic history of several common bipolar mosses; we examined whether their distributions result from recent inter-hemispheric dispersal events or long-term separation, and assessed the underlying drivers explaining their distributions. Our study focuses on four common bipolar species of Polytrichales, an old and distinct group of mosses, including three species from the genus *Polytrichum* Hedw. (*Polytrichum juniperinum* Hedw., *Polytrichum strictum* Brid. and *Polytrichum piliferum* Hedw.) plus one species of a closely related genus, *Polytrichastrum alpinum* Hedw. We particularly focused on *P. juniperinum*, due to its previously observed phenotypic variation throughout its global range [5]. All species occur in higher latitude areas in both hemispheres, with the bulk of their distributions in the NH. Their SH distributions are more restricted (in absolute area) to the Antarctic region (southern South America, the Atlantic sub-Antarctic islands and the Antarctic Peninsula), with some species having additional populations in other SH locations (figure 1; [5]). Although some of the species are sometimes described as cosmopolitan, according to the most recent global assessment [5], all are bipolar. *Polytrichum strictum* is strictly bipolar, whereas the other species also have restricted intermediate populations in high-altitude equatorial regions, a feature valuable for assessing whether these intermediate populations have acted as stepping-stones, are remnants of a once wider distribution (vicariance) or the result of separate colonization events.

## 2. Material and methods

### 2.1. Sampling and molecular methods

We sampled 71, 59, 73 and 52 individuals of *P. juniperinum*, *P. strictum, P. piliferum* and *P. alpinum*, respectively, representing their worldwide distributions (see electronic supplementary material, table S1 for sample information). Total genomic DNA (gDNA) was extracted using the DNeasy Plant Mini Kit (Qiagen GmbH, Hilden, Germany), using liquid nitrogen and a mortar and pestle. PCR amplification was performed using the Taq PCR Core Kit (Qiagen GmbH) with addition of bovine serum albumin, and results were checked using gel electrophoresis. Internal Transcribed Spacer (*ITS)* regions 1 (636–1007 bp) and 2 (386–441 bp) were amplified separately using primers ITS-A and ITS-C [11] for *ITS* 1, and 5.8S-R [12] and 25R [13], or ITS3 and ITS4 [14] for *ITS* 2. The plastid spacer *trnL-F* (455–545 bp) was amplified using primers *trn*LF-c and *trn*LF-f [15]. An annealing temperature of 60°C was used for all amplifications. Forward and reverse sequencing was performed by LGC Genomics (Berlin, Germany).

### 2.2. Sequence editing and alignment

Forward and reverse sequences were manually examined and assembled using Codoncode Aligner v. 5.0.2 (CodonCode Corp., Dedham, MA, USA). *ITS* and *trnL-F* sequences were aligned using PRANK [16] using default settings, with obvious misaligned sequences re-aligned manually. Short partially incomplete sections at the ends of each alignment were excluded. In the *trnL-F* fragment, a previously identified hairpin-associated inversion known to be highly homoplastic [17] was excluded. The *ITS* 1 and 2 fragments were combined, and hypervariable regions were identified and removed using NOISY [18] using default settings, resulting in a reduced alignment with 1614 bp (96.82% of the original 1667 bp). The number of variable and parsimony informative (PI) sites was calculated using MEGA7 [19].

### 2.3. Phylogenetic analyses

*Polytrichastrum tenellum* (Müll. Hal.) G.L. Sm. and *Meiotrichum lyallii* (Mitt.) G.L. Merr. (Genbank accessions GU569750 and AF545011, respectively) were chosen as outgroups for *trnL-F*. Based on the prior phylogenetic analyses [20,21] which have established sister-group relationships for this family, *P. alpinum* was used as an outgroup in the *ITS* 1 + 2 phylogenetic analyses.

Models of sequence evolution were selected for each locus using jModeltest-2.1.7 [22] using the SPR tree topology search operation and the Akaike information criterion (AICc). Maximum-likelihood (ML) analyses were performed for each locus using RAxML-GUI v. 1.3.1 [23], using GTR and GTR + G for *trnL-F* and *ITS* 1 + 2, respectively, applying default settings and estimating support values using 1000 bootstrap iterations. Bayesian analyses were performed for *trnL-F* and *ITS* 1 + 2 separately using MrBayes 3.2 [24], and were run for 1 × 10^6^ and 2 × 10^7^ million generations, respectively, sampled every 1.0 × 10^3^ generations, discarding the first 25% as burn-in. Convergence was assessed by checking split frequencies had an average standard deviation of less than 0.01, and by using Tracer v. 1.6 [25] to check all parameters had effective sample sizes greater than 200. Maximum clade credibility trees were visualized using FigTree v 1.4.2 (http://tree.bio.ed.ac.uk/software/figtree/).

### 2.4. Species delimitation

We explored possible species clusters in *ITS* 1 + 2 within the currently described species by testing for intraspecific divergence based on pairwise genetic distances using the Automatic Barcode Gap Discovery (ABGD) web server [26], using default settings. ABGD uses a genetic distance-based approach based on non-overlapping values of intra- and interspecific genetic distances, sorting the sequences into hypothetical candidate species.

### 2.5. Population diversity analyses

To examine the phylogeographical structure within species, TCS networks [27] were produced using *ITS* 1 + 2 for each species with Popart [28], using default settings. Because of a greater number of *ITS* 2 sequences available for *P. piliferum* and *P. alpinum*, we calculated additional haplotype networks for *ITS* 2 only for these species. Genetic diversity indices, pairwise Kimura-2P distances, demographic and spatial models and neutrality tests Tajima's *D* [29] and Fu's *F*s [30] were calculated for *ITS* 1 + 2 for each species with 10 000 permutations, using Arlequin v.3.5.1.2 [31]. We also performed these statistical analyses on various monophyletic clusters within *P. juniperinum*.

### 2.6. Molecular dating

Although *ITS* is a useful marker for investigating population- or species-level variation, it is too variable to be used directly in a larger dating analysis, including more distantly related species in which informative fossils can be incorporated. Therefore, to investigate the divergence times of the different species and populations, we used the following different calibration approaches:

(I) A two-step dating analysis consisting of:
  (I1) A larger Polytrichales dataset comprising markers *rbcL*, *trnL-F*, *rps4*, *rps4-trnS* and *nad5* [21]. We included the same fossil priors as in [21], and following [21], we performed analyses with (I1a) and without (I1b) the taxonomically uncertain fossil *Eopolytrichum antiquum* Konopka *et al*. [21,32,33]. For each analysis, we calculated the age of the split between *P. piliferum*/(*P. juniperinum* + *P. strictum*).
  (I2) The age (and 95% age distribution) of the node (*P. piliferum*/(*P. juniperinum* + *P. strictum*)) from step (I1) was applied as a secondary prior on the same node in the *ITS* 1 + 2 dataset. This was done for both ages resulting from step (I1) with (I1a) and without (I1b) the fossil *E. antiquum*, resulting in analyses I2a and I2b, respectively.
(II) A dating analysis based on a defined *ITS* substitution rate (1.35 × 10^−3^ subst. site Myr^−3^), previously applied in bryophytes [34,35], but originally derived from angiosperms ([36] and references therein).

For details regarding settings and priors in the BEAST analyses, see electronic supplementary material, S1 and figure S4.

### 2.7. Ancestral range distribution

We used the R-package BioGeoBEARS [37,38] to estimate the probabilities of ancestral area ranges at each node. This package estimates the maximum likelihood of the geographical range as well as models for evolution of geographical range along a time-calibrated phylogeny. We tested different models of dispersal, extinction and/or founder-event speciation (+J [38]) implemented in the script, selecting the best model using the AICc criterion and the likelihood ratio test. The maximum number of areas per node was set to five, the same as the number of regions specified in this study.

## 3. Results

### 3.1. Molecular sequence data

Samples were obtained from a broad range of locations for each species within their global distribution (for *ITS* 1 + 2 samples, figure 1; for *trnL-F* samples, see electronic supplementary material, figure S1). Alignments of *ITS* 1 + 2 and *trnL-F* consisted of 448–1007, 386–426 and 455–545 bp, respectively. The nuclear regions had more genetic variation (after treatment with NOISY [18]: *ITS* 1 = 244 and 220 variable and parsimony informative (PI) sites, respectively; *ITS* 2 = 72 and 66 variable and PI sites, respectively) than the *trnL-F* region (26 variable sites, 25 PI sites), reflecting the fact that *ITS* is faster evolving than *trnL-F*. Within *P. piliferum*, most intra-species variation occurred within a large indel (approx. 427 bp) which was found in *ITS* 1 and is unique to this species. AICc favoured the TPM3uf (nst = 6) model for *trnL-F* and TrN + G (nst = 6, rates = gamma) model for *ITS*1 + 2. A relatively high proportion of double peaks within *ITS* 1 + 2 chromatograms of several *P. strictum* specimens suggested multiple copies of *ITS* were present within some individuals of this species, possibly the result of a past hybridization event (see §3.3).

### 3.2. Phylogenetic relationships

**Figure 2.**
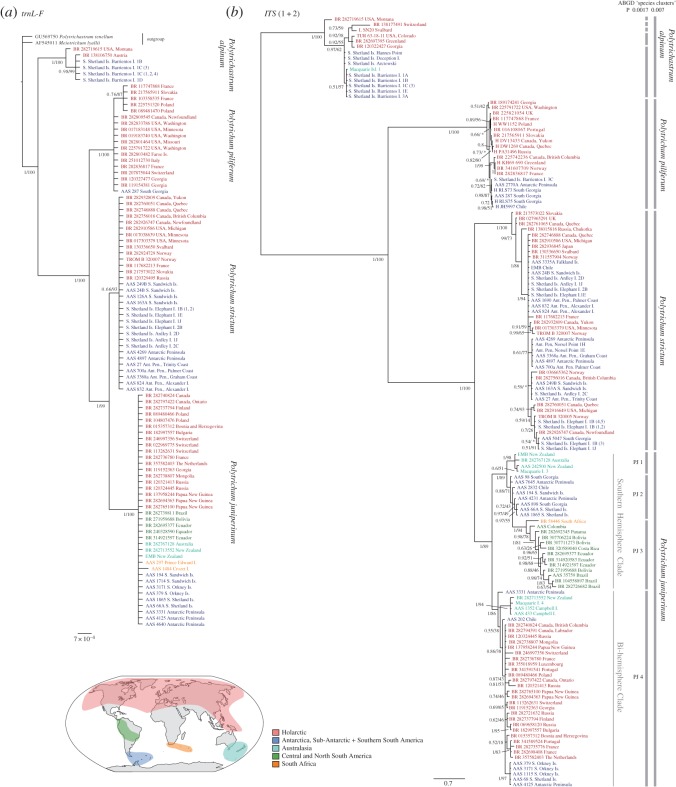
Bayesian phylogenies constructed with (*a*) plastid marker *trnL-F* and (*b*) nuclear marker *ITS* (1 + 2) for *P. alpinum, P. piliferum*, *P. strictum* and *P. juniperinum*. PP and bootstrap support are shown next to branches (*conflict between topologies of Bayesian and ML tree; see electronic supplementary material, figures S1 and S2 for ML phylogenies). Colours refer to different geographical regions (see map); outgroups are indicated in black. The scale bar represents the mean number of nucleotide substitutions per site. ABGD species delimitation clusters with different *p*_max_-values are shown in grey next to (*b*).

Bayesian phylogenetic trees based upon analyses of *trnL-F* and *ITS* 1 + 2 are shown in figure 2*a* and *b*, respectively, including posterior probabilities (PP) and bootstrap support (see electronic supplementary material, figures S2 and S3 for ML phylogenies of *trnL-F* and *ITS* 1 + 2, respectively). The phylogeny revealed a topology consistent with current species definitions and relationships [20,21]: *P. alpinum* was the most distantly related and, within *Polytrichum*, *P. piliferum* was more distantly related than the sister species *P. strictum* and *P. juniperinum.* No topological conflicts were found between Bayesian and ML analyses at key nodes (only PP are mentioned hereafter).

The *ITS* 1 + 2 tree (figure 2*b*) provided well-resolved clades with high support values (PP = 1.00) for all species. The *trnL-F* topology (figure 2*a*) showed high support values (PP = 1.00) for all species except for *P. strictum* (PP = 0.66). The species delimitation method ABGD [26] revealed two significant ‘barcoding gaps’ in *ITS* 1 + 2 at Prior ‘maximum divergence of intraspecific diversity’ (*p*_max_) values 0.0017 and 0.0077 (figure 2). At *p*_max_ = 0.0077, four groups were identified, consistent with current morphological species definitions. At *p*_max_=0.0017, five and four distinct groups were identified within *P. alpinum* and *P. juniperinum*, respectively, suggesting greater phylogenetic structure within these two species than is currently recognized taxonomically.

### 3.3. Biogeographic patterns within species

**Figure 3.**
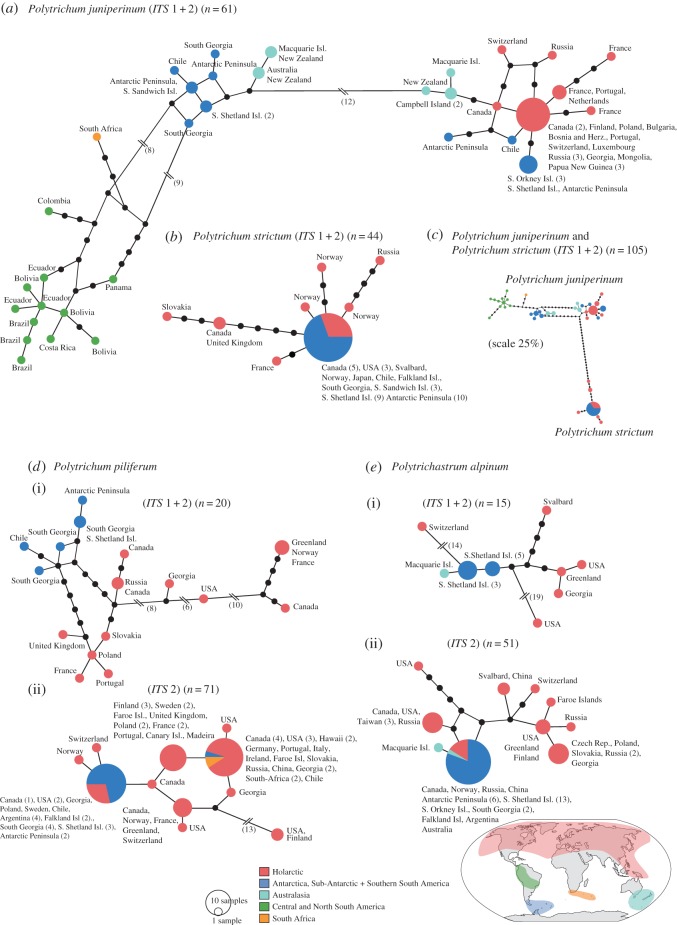
Haplotype network of *ITS* 1 + 2 of (*a*) *P. juniperinum*, (*b*) *P. strictum*, (*c*) sister species *P. juniperinum* and *P. strictum* together, (*d*) *P. piliferum* and (*e*) *P. alpinum*. Separate haplotype networks of *ITS* 1 + 2 ((*d*,*e*) (i)) and *ITS* 2 only ((*d*,*e*)(ii)) are shown for the last two species. Haplotype circle sizes correspond to numbers of individuals with the same haplotype (see legend). Branches represent mutations between haplotypes, with mutations shown as one-step edges or as numbers. Colours refer to the different geographical regions (see map).

Biogeographic patterns within species were interpreted based on the phylogenetic tree topologies (*trnL-F* and *ITS* 1 + 2; figure 2) and structure of haplotype networks (*ITS* 1 + 2 and *ITS* 2; figure 3).

Within *P. juniperinum*, two strongly supported clades (PP = 1.00) with different geographical distributions were apparent in the *ITS* 1 + 2 topology (figure 2*b*). The first clade consisted of SH regions (hereafter the ‘SH clade’) with several subclades: a monophyletic Australasian subclade (PP = 1.00; ABGD-cluster PJ 1), a monophyletic subclade including one South African specimen and several low-latitude South American specimens (PP = 1.00; ABGD-cluster PJ 3) and a non-monophyletic group of lineages including specimens from Antarctica, the sub-Antarctic and southern South America (with PP varying from 0.72 to 0.97; defined by ABGD as cluster PJ 2). The second clade (hereafter the ‘bi-hemispheric’ clade) included multiple early-diverging lineages from the SH (including Australasia and the Antarctic region; PP = 0.55–1.00), and a large monophyletic subclade (PP = 0.87) with specimens from the NH as well as a distinct monophyletic group composed of Antarctic and sub-Antarctic specimens (PP = 1.00). A similar pattern was apparent in the haplotype network (figure 3*a*), where the two main (‘SH’ and ‘bi-hemispheric’) clades diverged by 12 mutational steps, with SH specimens on either side.

In contrast with *P. juniperinum*, the *ITS* 1 + 2 region within *P. strictum* revealed multiple chromatogram peaks in multiple samples, probably due to a duplication of *ITS* within the species. As ambiguous positions are not taken into account in the phylogenetic or haplotype analyses, this resulted in an underrepresentation of the genetic variation, and no strong biogeographic patterns could be inferred (figure 2*b*). However, we found that, despite the exclusion of ambiguous sites, several NH specimens were placed as sister groups to a monophyletic clade of all other (NH and SH) specimens (figure 2*b*). The *P. strictum* haplotype network revealed the highest genetic variation in NH specimens, showing a single haplotype including both SH and NH specimens, with several NH haplotypes diverging by one to six mutational steps from the main haplotype (figure 3*b*). We further explored the genetic diversity within *P. strictum* by phasing ambiguous haplotypes into different haplotypes within individuals using Phase v. 2.1.1 [39,40], applying default options, followed by the same downstream analyses; however this did not improve phylogenetic resolution (data not shown). Although the phylogeographical history of *P. strictum* needs further assessment, the phenomenon of multiple chromatogram peaks is noteworthy in itself, possibly representing the second known case of *ITS* paralogy in mosses [41]. The phenomenon is possibly the result of a past hybridization event in *P. strictum*, as previously suggested by Bell & Hyvönen [20]. Similar patterns were not observed in the other study species.

As a greater number of *ITS* 2 sequences were available in *P. piliferum* and *P. alpinum*, we analysed *ITS* 1 + 2 and *ITS* 2 in separate haplotype networks for these species (figure 3*d*,*e*). As described, most intra-species variation in *P. piliferum* is located in a 427 bp insertion, which was not found in the other species. This diversity was therefore masked when it was aligned with the other species for analysis in a Bayesian phylogenetic framework (figure 2). The *ITS* 1 + 2 phylogeny (figure 2*b*) revealed three weakly resolved monophyletic clusters of NH specimens and placed all SH specimens together in a fourth monophyletic group. Similarly, the *ITS* 1 + 2 and *ITS* 2 networks of *P. piliferum* (figure 3*d*) revealed multiple clusters, with most genetic variation found between NH specimens. In the *ITS* 1 + 2 haplotype network (figure 3*d*(i)), all SH haplotypes clustered closely together. The *ITS* 2-only haplotype network (figure 3*d*(ii)) showed several distinct haplotypes. One of the main haplotypes included individuals from the NH and most SH individuals, including all Antarctic and sub-Antarctic specimens. However, a separate, common haplotype included individuals of the NH as well as a specimen from Chile and two from South Africa.

The *ITS* 1 + 2 phylogeny of *P. alpinum* revealed one genetically divergent NH specimen from North America at the base of the clade. Remaining specimens were broadly clustered into SH and NH groups, with the NH group being monophyletic and containing more phylogenetic structure than the paraphyletic cluster of SH specimens (figure 2*b*). A greater diversity of NH haplotypes was also found in both *ITS* 1 + 2 and *ITS* 2 networks (figure 3*e*), which revealed distinct regional NH clusters, while all SH specimens were grouped closely together.

### 3.4. Population expansion analyses

Population expansion and neutrality tests to infer the demographic history of each species, as well as particular monophyletic and ABGD-defined clusters (figure 2*b*) within *P. juniperinum*, are shown in electronic supplementary material, table S2*.* Demographic and spatial expansion tests did not reject a null hypothesis of population expansion for any species or population within *P. juniperinum* (all *p*-values were non-significant), supporting possible demographic and spatial expansion in all clusters. An excess of low-frequency polymorphisms over that expected under neutrality was inferred from significantly negative Fu's *F*s values [30] for all groups within *P. juniperinum*. Considering these data in relation to the haplotype network patterns, the species clade(s) most likely to reflect a past population expansion are the *P. juniperinum* ‘bi-hemisphere clade’ and/or ‘Holarctic + recent Antarctic dispersal event’ clades, which have significant Fu's *F*s and large negative Tajima's *D* values (and low though non-significant *p*-values) and a star-shaped haplotype network topology. Mismatch distribution patterns are also consistent with this possibility (see electronic supplementary material, figure S5). Another species reflecting a possible past expansion was *P. alpinum*, the only species with a significant and large negative Tajima's *D* value.

### 3.5. Geographical range probabilities and molecular dating

**Figure 4.**
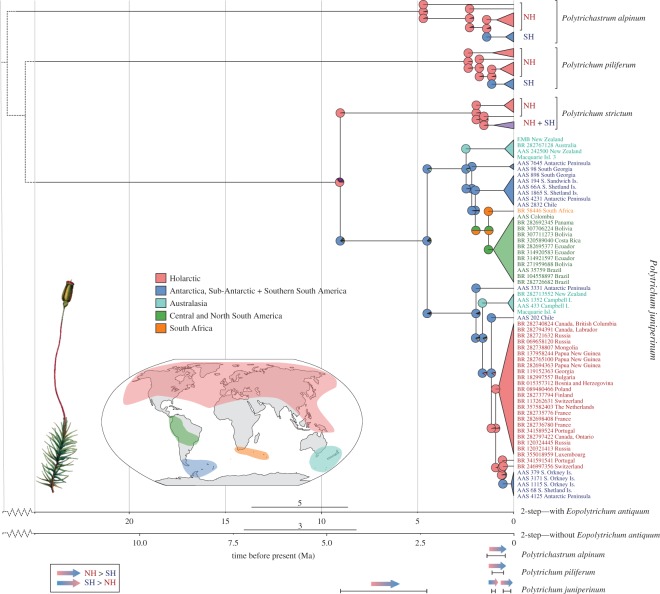
Historical biogeography of four Antarctic Polytrichaceae mosses, highlighting the population history of *P. juniperinum*. The maximum clade credibility tree shows the median divergence time estimates calculated with two two-step dating analyses, with (*a*) or without (*b*) including the taxonomically uncertain fossil *E. antiquum* as a prior. Median ages and 95% height posterior distributions associated with major nodes are presented in electronic supplementary material, table S3. Coloured pie-charts represent ancestral range probabilities at each node as recovered by the best BioGeoBEARS model. Colours refer to the different geographical regions (see map). Arrows below the figure represent the time and direction of inter-hemispheric movements of all species excluding *P. strictum*. NH and SH represent Northern and Southern Hemispheres, respectively. The black line below each arrow is the branch, and therefore, timeframe over which the inter-hemispheric movement (according to ancestral range probabilities) was estimated to have occurred (note that 95% height posterior distribution of these branches is not presented here).

The ancestral range estimates and molecular dating provided estimates of the diversification, timing and spatial origins of the inter-hemispheric distribution in each species (figure 4). The ancestral range estimates under the R-program BioGeoBEARS [38] selected the DEC + J model of species evolution (dispersal–extinction–cladogenesis (DEC), implementing a founder-effect component (+J) [38]). The ancestral area reconstruction suggested the earliest lineages within *P. alpinum*, *P. piliferum* and the ancestor of *P. strictum* and *P. juniperinum* were of Holarctic origin (figure 4), and that their SH populations were the result of NH to SH movements. However, as *P. strictum* and *P. juniperinum* diverged, while the ancestor of *P. strictum* remained in the Holarctic for several million years further, the ancestor of *P. juniperinum* dispersed to the Antarctic region (Antarctic, sub-Antarctic and/or southern S. America). From here, *P. juniperinum* diverged into two different clades, and dispersed into Australasia (from both clades), South Africa and low-latitude regions in South America, as well as the entire Holarctic region. Subsequently, a separate trans-equatorial dispersal event occurred from the Holarctic back to the Antarctic region.

Results of all dating analyses are shown in electronic supplementary material, table S3 and figure 4 (only showing two-step dating analyses with (I2a) and without (I2b) the fossil *E. antiquum*). Applying the nuclear rate (Method II) resulted in considerably older age estimates than those using the two-step approach (Method I), with ages almost three times greater than those of the oldest two-step approach (I2a; including the fossil). Following the dating analysis that provides the most recent divergence time estimates (I2b; without *E. antiquum*), all SH migrations occurred within the Pleistocene (*P. alpinum*, *P. piliferum*), Pliocene/Early Pleistocene (initial SH arrival *P. juniperinum*) and Pleistocene (recent Holarctic to Antarctic dispersal within *P. juniperinum*). Ages calculated under I2a (with *E. antiquum*) suggested SH migrations to have occurred during the Pleistocene (*P. alpinum, P. piliferum*), Late Miocene/Early Pliocene (initial SH arrival *P. juniperinum*) and Pleistocene (recent Holarctic to Antarctic dispersal within *P. juniperinum*). Following the rate analysis (Method II), SH migrations occurred within the Late Pliocene/Pleistocene (*P. alpinum*, *P. piliferum*), Late Oligocene/Early Miocene (initial SH arrival *P. juniperinum*) and Pleistocene (recent Holarctic to Antarctic dispersal within *P. juniperinum*).

## 4. Discussion

### 4.1. Long-distance dispersal as driver of species-level disjunctions

Even with the differences between the various dating analyses, all analyses identified similar outcomes indicating that the main inter-hemispheric movements occurred on hundred-thousand to multi-million-year timescales, from the Pleistocene, Pliocene and/or Miocene/Late Oligocene. Divergence times as calculated here are likely to be underestimated, as there is evidence that substitution rates of mosses are considerably lower than in vascular plants [42], suggesting that even estimations under the rate analysis (Method II) may be too recent, and thus divergence events may have occurred further back in time. Nevertheless, even if underestimated, divergence times between populations are too young to have derived from continental vicariance (e.g. looking at southern landmasses: New Zealand, Australia and South America became separated from Antarctica approx. 80 Ma, approx. 35–30 Ma and approx. 30–28 Ma, respectively [43]). Additionally, a situation where North and South American populations were the result of a connection via the Isthmus of Panama (approx. 15 Ma [44]) is also not supported by the direction of migration (i.e. northern South American populations of *P. juniperinum* did not act as ‘stepping-stones’ but were derived from separate migrations). It could be that bipolar populations have derived from climatic vicariance (e.g. temperate populations became separated by unfavourable conditions across the tropics); however, we find the inter-hemispheric dispersal events have occurred over much longer timescales than might be expected had such events been associated with, for example, the last major glaciation (i.e. the Last Glacial Maximum; LGM). Our results therefore support the hypothesis that long-distance dispersal is the underlying driver for the bipolar disjunctions considered in these species.

Such long-distance dispersal could have taken place via spores (generally less than 10 µm [5,45] in these genera) or other propagules, either via wind currents or animal vectors, such as migratory birds. The patterns observed here clearly illustrate the dispersal abilities of bryophytes yet, even so, major trans-equator dispersal events have been extremely rare. In *P. juniperinum*, successful inter-hemispheric movements appear to have occurred only three times: first, at the split which separated *P. juniperinum* from the ancestor of *P. strictum + P. juniperinum*; second, the SH to NH dispersal event and third, the final and much more recent NH to SH migration. In *P. piliferum*, two or more independent trans-equatorial dispersal events occurred (figure 3*d*(ii): SH specimens found in two separate clusters; Antarctic/sub-Antarctic in one, South African in one and Chilean in both). In *P. alpinum*, all SH specimens were clustered closely together, suggesting just one NH to SH dispersal event; however, sampling is more limited for this species. Trans-equatorial dispersals occurred from north to south in all species, and from south to north in *P. juniperinum*. Analyses of *P. strictum* also revealed higher levels of genetic variation in the NH than the SH, with biogeographic patterns indicating that the species probably originated in the NH, and subsequently dispersed to the SH.

It should be noted that the biogeographic and population genetic patterns here are inferred based largely on the variation in the *ITS* region, a marker widely applied in plant phylogenetics and population genetics [46]. However, multiple copies have been found within the genome for some species [41,46], as probably observed here in *P. strictum,* which can complicate the interpretation of biogeographic patterns with this marker. Further analysis with additional markers would enhance our understanding of these population genetic patterns.

### 4.2. Within-species variation in *Polytrichum juniperinum* and *Polytrichastrum alpinum*

The species delimitation analysis identified several clusters within *P. juniperinum* and *P. alpinum*, with genetic differentiation consistent with species-level differentiation (figure 2). Both species are known to be phenotypically variable throughout their range, prompting classification of several infraspecific taxa or subspecies (*P. juniperinum* [47,48] and *P. alpinum* ([5] and references therein; [49]). Although not currently recognized through assumed phenotypic plasticity [5], these distinctions regain credence here based on the variability in the *ITS* region. How genetic variation in *P. juniperinum* and *P. alpinum* is correlated with phenotypic variation and whether the species' current classifications should encompass several subspecies or taxa of higher status requires further study integrating morphological and genetic approaches.

### 4.3. No ‘stepping-stone’ dispersal in *Polytrichum juniperinum*

We found no evidence that intermediate high-elevation populations in the South American tropics or South Africa in *P. juniperinum* have acted as ‘stepping-stones’ for inter-hemispheric dispersal. Rather, these intermediate populations appear to be the result of separate northwards colonization events from the Antarctic region. Such northward movements could have been facilitated by a temporary lowering of vegetation zones and treelines during interglacial periods, as has been suggested as a mechanism to explain the presence of several members of Polytrichaceae in high-elevation areas in tropical South America (e.g. *Polytrichadelphus* Müll. Hal (Mitt.) [8]). Genetic evidence for northward dispersal into the lower latitudes of South America has only been reported before in five families of angiosperm [50], and once in a hornwort genus [51], but never before in mosses.

### 4.4. Dispersal out of the Antarctic region

Phylogeographical analyses suggest all the contemporary and disjunct populations within *P. juniperinum* originated through dispersal from the Antarctic region, including populations in the South American tropics and South Africa, Australasia and the Holarctic (figure 4). Two separate migrations from the Antarctic region to Australasia were apparent, revealing a relatively strong connection between these regions, possibly assisted by the strong circumpolar ‘westerly wind’ belt, a link also implied in an SH aerobiology modelling study [52] and descriptions of bryophyte biogeographic regions [53]. Very little differentiation was identified across the NH distribution of *P. juniperinum* (‘Holarctic + recent Antarctic dispersal event’ clade; see electronic supplementary material, table S2 and figure S5). This, together with a significantly negative Fu's *F*s in this clade and star-like haplotype network, suggests a rapid NH colonization from a single or limited number of northward dispersal events from the SH. Favourable conditions for this could have been facilitated by the harsh Pleistocene glacial periods in the Holarctic, which, on ice retreat, left extensive barren areas available for colonization for cold-adapted mosses [8].

### 4.5. Persistence in Southern Hemisphere glaciated regions

Our divergence time analyses imply *P. juniperinum, P. alpinum* and *P. piliferum* all arrived in the Antarctic, sub-Antarctic and/or southern South America well before the LGM. All these regions are thought to have experienced extensive glaciations throughout the LGM and previous glacial cycles, although biological evidence supports the existence of glacial refugia in both southern South America [54] and the Antarctic [55–57]. Whether Antarctic and sub-Antarctic populations of our study species are of recent (post-LGM) origin through repeated dispersal events from southern South America, or have persisted in the far south *in situ* requires further investigation.

Recent modelling studies [58,59] have highlighted considerably greater dynamism in ice extent throughout glacial cycles in the Antarctic Peninsula region over the timescales of interest here than has previously been suspected. Several warmer-than-present interglacials occurred throughout the Pleistocene [58–61] and Early Pliocene [62], while the increased dynamism apparent in these models may provide a foundation allowing the persistence of previously unconsidered ice-free regional refugial areas. Additionally, both *P. juniperinum* and *P. alpinum* can often be found growing in geothermally influenced areas on volcanic Antarctic and sub-Antarctic islands [5,63], which are suggested as possible regional refugia [64]. Furthermore, recent studies of polar mosses have shown subglacial or within permafrost survival over several hundred years [65] to millennial timescales [66]. Although such timescales still fall short of those required for persistence through entire glacial cycles, these studies suggest that mosses have the potential to survive through at least shorter periods (several centuries) of ice expansion, and possibly longer periods of unfavourable conditions. Recently, the weedy, cosmopolitan moss *Bryum argenteum* Hedw. was suggested to have a multi-million-year Antarctic persistence [67], providing a first intriguing suggestion that long-term persistence might be a more general feature of today's Antarctic flora, and one that is at least consistent with the data presented in this study.

## Supplementary Material

Biersma et al ESM

## Data accessibility

DNA sequences (as listed in electronic supplementary material, table S1): Genbank accessions MF180301-MF180632. Data available on the Dryad Digital Repository (http://dx.doi.org/10.5061/dryad.4m35m) [68].
