# Supplementary material for: Global biogeographic patterns in bipolar moss species
Source: R Soc Open Sci. 2017 Jul 12;4(7):170147. doi: 10.1098/rsos.170147 (PMC5541534; doi:10.1098/rsos.170147)
Supplement: Biersma et al ESM [file rsos170147supp1.docx]

**Electronic supplemental materials**

**Sample information:**

Samples originated from herbaria at the British Antarctic Survey (AAS), the Botanic Garden in Meise (BR), the Finnish Museum of Natural History (H), the University of Turku (TUR), the University of Tromsø (TROM), the National Herbarium of the Netherlands (L) and the Bolus Herbarium, South Africa (BOL), and expeditions of authors EB and PC.

**Table S1**. Specimens used in this study including herbarium details, accession numbers, geographic locations and GenBank accession numbers. “Antarctic region” stands for Antarctic, sub-Antarctic and southern South America. Brackets behind specific specimens represent multiple stems being taken from within ~5 cm, which could have represented the same individual and have been treated as one if the sequence was identical. Samples of ITS2 only of *Polytrichum strictum* have not been analysed in this study. - = not available

| Specimen name | Geographic origin (country) | Geographic region as used in this study | Herbarium + herbarium number (when applicable) | Collection + collection number (when applicable) | Latitude and longitude (decimal degr.) | *ITS* 1 + 2 | *ITS* 2 | *trnL-F* |
| --- | --- | --- | --- | --- | --- | --- | --- | --- |
| *Polytrichum juniperinum* |  |  |  |  |  |  |  |  |
| BR 282794391 Canada, Labrador | Canada | Holarctic | BR 5040282794391 | G.R. Brassard | 53.6, -64.32 | MF180399 | - | - |
| BR 282740824 Canada, British Columbia | Canada | Holarctic | BR 5040282740824 | D.H. Vitt | 52.11, -119.65 ^1^ | MF180400 | - | MF180539 |
| BR 282797422 Canada, Ontario | Canada | Holarctic | BR 5040282797422 | B. Allen | 48.77, -88.63 ^1^ | MF180401 | - | MF180540 |
| BR 069658120 Russia | Russia | Holarctic | BR 5040069658120 | V. Vašák | 43.4, 42.92 ^1^ | MF180402 | - | - |
| BR 282698408 France | France | Holarctic | BR 5040282698408 | J.L. De Sloover | 44.89, -1.21 ^1^ | MF180403 | - | - |
| BR 282721632 Russia | Russia | Holarctic | BR 5040282721632 | I. Dabkowska | 55.8, 37.68 ^1^ | MF180404 | - | - |
| BR 282735776 France | France | Holarctic | BR 5040282735776 | L. Leclercq | 50.81, 1.61 ^1^ | MF180405 | - | - |
| BR 341589524 Portugal | Portugal | Holarctic | BR 5040341589524 | T. Arts | 32.73, -16.89 ^1^ | MF180406 | - | - |
| BR 341591541 Portugal | Portugal | Holarctic | BR 5040341591541 | T. Arts | 32.76, -17.08 ^1^ | MF180407 | - | - |
| BR 355018959 Luxembourg | Luxembourg | Holarctic | BR 5040355018959 | E. Jacques | 49.91, 6.06 | MF180408 | - | - |
| BR 015357312 Bosnia and Herzegovina | Bosnia and Herzeg. | Holarctic | BR 5040015357312 | A. Rusinska | 43.57, 17.43 ^2^ | MF180409 | - | MF180541 |
| BR 089480466 Poland | Poland | Holarctic | BR 5040089480466 | S. Lisowski, A. Rusinska & I. Melosik | 50.83, 23.4 ^1^ | MF180410 | - | MF180542 |
| BR 182997557 Bulgaria | Bulgaria | Holarctic | BR 5040182997557 | V. Vašák | 41.77, 23.4 ^1^ | MF180411 | - | MF180543 |
| BR 246997356 Switzerland | Switzerland | Holarctic | BR 5040246997356 | M. Onraedt | 46.19, 7.63 ^1^ | MF180412 | - | MF180544 |
| BR 282736780 France | France | Holarctic | BR 5040282736780 | J.L. De Sloover | 44.04, -1.34 ^1^ | MF180413 | - | MF180545 |
| BR 282737794 Finland | Finland | Holarctic | BR 5040282737794 | T. Lammes | 60.45, 22.11 ^1^ | MF180414 | - | MF180546 |
| BR 357582403 Netherlands | Netherlands | Holarctic | BR 5040357582403 | J.A.E. Slembrouck | 51.42, 4.8 ^1^ | MF180415 | - | MF180547 |
| BR 113262631 Switzerland | Switzerland | Holarctic | BR 5040113262631 | A. Lawalrée | 46.14, 7.01 | MF180416 | - | MF180548 |
| BR 104807476 Poland | Poland | Holarctic | BR 5040104807476 | H. Bednarek-Ochyra & R. Ochyra | 53.95, 22.3 ^1^ | - | - | MF180549 |
| BR 022969775 Switzerland | Switzerland | Holarctic | BR 5040022969775 | A. Lawalrée | 46, 7.34 ^1^ | - | - | MF180550 |
| BR 119152363 Georgia | Georgia | Holarctic | BR 5040119152363 | V. Vašák | 42.4, 43.94 ^1^ | MF180417 | - | MF180551 |
| BR 120321413 Russia | Russia | Holarctic | BR 5040120321413 | V. Vašák | 51.66, 103.7 ^1^ | MF180418 | - | MF180552 |
| BR 120324445 Russia | Russia | Holarctic | BR 5040120324445 | V. Vašák | 56.31, 101.66 ^1^ | MF180419 | - | MF180553 |
| BR 137958244 Papua New Guinea | Papua New Guinea | Holarctic | BR 5040137958244 | J.L. De Sloover | -6.04, 143.89 ^1^ | MF180420 | - | MF180554 |
| BR 282694363 Papua New Guinea | Papua New Guinea | Holarctic | BR 5040282694363 | J.L. De Sloover | -5.83, 143.4 ^1^ | MF180421 | - | MF180555 |
| BR 282738807 Mongolia | Mongolia | Holarctic | BR 5040282738807 | A. Pacyna | 47.5, 100 ^1^ | MF180422 | - | MF180556 |
| BR 282765100 Papua New Guinea | Papua New Guinea | Holarctic | BR 5040282765100 | J.F. Veldkamp & M. Kuduk | -8.21, 146.78 | MF180423 | - | MF180557 |
| BR 58446 South Africa | South Africa | S. African | BR 040058446523 | E. Powrie | -34.06, 19.53 ^1^ | MF180424 | - | - |
| AAS 1484 Crozet I. | Crozet I. | S. African | AAS | B.G. Bell 01484 | -46.42, 51.83 | - | - | MF180558 |
| AAS 297 Prince Edward I. | Prince Edward I. | S. African | AAS | N.J.M. Gremmen 00297 | -46.92, 37.75 | - | - | MF180559 |
| AAS 242500 New Zealand | New Zealand | Australasia | AAS | E.M. Chapman s.n. | -42.38, 172.4 ^1^ | MF180425 | - | - |
| BR 282713552 New Zealand | New Zealand | Australasia | BR 5040282713552 | s.n. | -45.54, 169.31 ^1^ | MF180426 | - | MF180560 |
| BR 282767128 Australia | Australia | Australasia | BR 5040282767128 | H. Streimann | -37.07, 149.47 | MF180427 | - | MF180561 |
| EMB New Zealand | New Zealand | Australasia | BAS dried samples | E.M. Biersma s.n. | -45.53, 167.86 ^1^ | MF180428 | - | MF180562 |
| Macquarie I. 3 | Macquarie I. | Australasia | BAS frozen samples | D. Hodgson s.n. | -54.62, 158.86 ^1^ | MF180429 | - | - |
| Macquarie I. 4 | Macquarie I. | Australasia | BAS frozen samples | D. Hodgson s.n. | -54.62, 158.86 ^1^ | MF180430 | - | - |
| AAS 1352 Campbell I. | Campbell I. | Australasia | AAS | D.H. Vitt | -52.55, 169.15 | MF180431 | - | - |
| AAS 433 Campbell I. | Campbell I. | Australasia | AAS | R.L. Oliver 00433 | -52.55, 169.15 | MF180432 | - | - |
| BR 104558897 Brazil | Brazil | South America | BR 5040104558897 | V. Nicolack & O.S. Ribas | -25.3, -49.06 ^1^ | MF180433 | - | - |
| BR 282692345 Panama | Panama | South America | BR 5040282692345 | M. Nee | 8.87, -82.58 ^1^ | MF180434 | - | - |
| BR 282726682 Brazil | Brazil | South America | BR 5040282726682 | H.S. Irwin, E. Onishi, S.F. Da Fonseca, R. Souza, R. Reis Dos Santos & J. Ramos | -18.08, -43.74 ^1^ | MF180435 | - | - |
| BR 307706224 Bolivia | Bolivia | South America | BR 5040307706224 | I.G. Vargas | -18.72, -64.02 | MF180436 | - | - |
| BR 307711273 Bolivia | Bolivia | South America | BR 5040307711273 | I.G. Vargas | -17.82, -64.62 | MF180437 | - | - |
| BR 314920583 Ecuador | Ecuador | South America | BR 5040314920583 | T. Arts | -0.46, -78.45 ^1^ | MF180438 | - | - |
| BR 320589040 Costa Rica | Costa Rica | South America | BR 5040320589040 | T. Arts | 9.56, -83.8 ^1^ | MF180439 | - | - |
| AAS 35759 Brazil | Brazil | South America | AAS | W.R. Anderson, M. Stieber & J.H. Kirkbride Jr. s.n. | -18.4, -43.35 ^2^ | MF180440 | - | - |
| AAS Colombia | Colombia | South America | AAS | C.E. Chardón Palacios s.n. | 5.3, -75.25 ^1^ | MF180441 | - | - |
| BR 271959688 Bolivia | Bolivia | South America | BR 5040271959688 | M. Nee | -18.07, -63.92 | MF180442 | - | MF180563 |
| BR 282695377 Ecuador | Ecuador | South America | BR 5040282695377 | L.J. Dorr & L.C. Barnett | -2.88, -78.77 | MF180443 | - | MF180564 |
| BR 314921597 Ecuador | Ecuador | South America | BR 5040314921597 | T. Arts | -0.68, -78.44 | MF180444 | - | MF180565 |
| BR 240328590 Ecuador | Ecuador | South America | BR 5040240328590 | M. Lewis | 0.37, -78.33 | - | - | MF180566 |
| BR 282739811 Brazil | Brazil | South America | BR 5040282739811 | J.-P. Frahm | -23.86, -46.21 ^1^ | - | - | MF180567 |
| AAS 1115 S. Orkney Is. | S. Orkney Is. | Antarctic region | AAS | R.E. Longton | -60.7, -45.67 | MF180445 | - | - |
| AAS 898 S. Georgia | South Georgia | Antarctic region | AAS | B.G. Bell 00898 | -54.5, -36.17 | MF180446 | - | - |
| AAS 98 South Georgia | South Georgia | Antarctic region | AAS | S.W. Greene 00098 | -54.23, -36.52 | MF180447 | - | - |
| AAS 194 S. Sandwich Is. | S. Sandwich Is. | Antarctic region | AAS | P. Convey 00194 | -57.07, -26.7 | MF180448 | - | MF180568 |
| AAS 379 S. Orkney Is. | S. Orkney Is. | Antarctic region | AAS | R.I.L. Smith 00379 | -60.7, -45.67 | MF180449 | - | MF180569 |
| AAS 1714 S. Sandwich Is. | S. Sandwich Is. | Antarctic region | AAS | R. Ochyra 01714 | -62.08, -58.25 | - | - | MF180570 |
| AAS 202 Chile | Chile | Antarctic region | AAS | S.W. Greene | -50.68, -72.37 | MF180450 | - | - |
| AAS 2832 Chile | Chile | Antarctic region | AAS | C.M. Matteri | -51.4, -73.07 | MF180451 | - | - |
| AAS 4231 Antarctic Peninsula | Antarctic Peninsula | Antarctic region | AAS | R.I.L. Smith 04231 | -64.73, -62.55 | MF180452 | - | - |
| AAS 68 S. Shetland Is. | S. Shetland Is. | Antarctic region | AAS | R.I.L. Smith 00068 | -62.98, -60.58 | MF180453 | - | - |
| AAS 7645 Antarctic Peninsula | Antarctic Peninsula | Antarctic region | AAS | R.I.L. Smith 07645 | -63.93, -57.82 | MF180454 | - | - |
| AAS 1865 S. Shetland Is. | S. Shetland Is. | Antarctic region | AAS | R. Ochyra 01865 | -62.17, -58.48 | MF180455 | - | MF180571 |
| AAS 3171 S. Orkney Is. | S. Orkney Is. | Antarctic region | AAS | R.I.L. Smith 03171 | -60.7, -45.67 | MF180456 | - | MF180572 |
| AAS 3331 Antarctic Peninsula | Antarctic Peninsula | Antarctic region | AAS | R.I.L. Smith 03331 | -65.42, -64.23 | MF180457 | - | MF180573 |
| AAS 4125 Antarctic Peninsula | Antarctic Peninsula | Antarctic region | AAS | R.I.L. Smith 04125 | -64.33, -62.93 | MF180458 | - | MF180574 |
| AAS 66A S. Shetland Is. | S. Shetland Is. | Antarctic region | AAS | D. Mason 00066A | -62.98, -60.58 | MF180459 | - | MF180575 |
| AAS 4640 Antarctic Peninsula | Antarctic Peninsula | Antarctic region | AAS | R.I.L. Smith 4640 | -67.97, -67.32 | - | - | MF180576 |
| *Polytrichum strictum* |  |  |  |  |  |  |  |  |
| BR 282761065 Canada, Quebec | Canada | Holarctic | BR 5040282761065 | J.L. De Sloover | 47.42, -61.76 ^1^ | MF180460 | - | - |
| BR 282746888 Canada, Quebec | Canada | Holarctic | BR 5040282746888 | J.L. De Sloover | 47.84, -69.53 ^1^ | MF180461 | - | MF180577 |
| BR 282759048 Canada, Quebec | Canada | Holarctic | BR 5040282759048 | J.L. De Sloover | 47.38, -61.87 ^1^ | - | MF180301 | - |
| BR 282760051 Canada, Quebec | Canada | Holarctic | BR 5040282760051 | J.L. De Sloover | 47.25, -61.93 ^1^ | MF180462 | - | MF180578 |
| BR 282926747, Canada, Newfoundland | Canada | Holarctic | BR 5040282926747 | G.R. Brassard | 55.26, -59.65 ^1^ | MF180463 | - | MF180579 |
| BR 282932809 Canada, Yukon | Canada | Holarctic | BR 5040282932809 | D.H. Vitt | 64.28, -140.45 | MF180464 | - | MF180580 |
| BR 282756016 Canada, British Columbia | Canada | Holarctic | BR 5040282756016 | D.H. Norris | 52.55, -125.72 | MF180465 | - | MF180581 |
| BR 089717890 U.S.A., Alaska | U.S.A. | Holarctic | BR 5040089717890 | P. Alpert | 66.36, -147.39 ^1^ | - | MF180302 | - |
| BR 282916649 U.S.A., Michigan | U.S.A. | Holarctic | BR 5040282916649 | H. Crum & N.G. Miller | 45.61, -84.73 ^1^ | MF180466 | - | - |
| BR 282910586 U.S.A., Michigan | U.S.A. | Holarctic | BR 5040282910586 | J. Jaworski | 42.4, -84.22 ^1^ | MF180467 | - | MF180582 |
| BR 017303379 U.S.A., Minnesota | U.S.A. | Holarctic | BR 5040017303379 | J.A. Janssens | 47.23, -94.95 | MF180468 | - | MF180583 |
| BR 017038639 U.S.A., Minnesota | U.S.A. | Holarctic | BR 5040017038639 | J.A. Janssens | 48.34, -94.54 | - | - | MF180584 |
| BR 027965291 United Kingdom | United Kingdom | Holarctic | BR 5040027965291 | S.L. Jury & F.J. Rumsey | 51.88, -3.7 | MF180469 | - | - |
| BR 036605362 Norway | Norway | Holarctic | BR 5040036605362 | R.E. Longton | 71.05, 28.02 ^1^ | MF180470 | - | - |
| BR 311557904 Norway | Norway | Holarctic | BR 5040311557904 | T. Arts | 69.27, 20.01 ^1^ | MF180471 | - | - |
| TROM B 320005 Norway | Norway | Holarctic | TROM B 320005 | A. Elvebakk & A.A. Frisvoll s.n. | 69.27, 20.52 ^1^ | MF180472 | - | - |
| TROM B 320007 Norway | Norway | Holarctic | TROM B 320007 | A. Sortland s.n. | 69.1, 18.05 ^1^ | MF180473 | - | MF180585 |
| BR 282924729 Norway | Norway | Holarctic | BR 5040282924729 | R. Alava | 69.58, 20.16 ^1^ | - | - | MF180586 |
| BR 217573022 Slovakia | Slovakia | Holarctic | BR 5040217573022 | J. Bouharmont | 49.17, 20.08 ^1^ | MF180474 | - | MF180587 |
| BR 117682213 France | France | Holarctic | BR 5040117682213 | J.-P. Frahm | 47.94, 6.59 ^1^ | MF180475 | - | MF180588 |
| BR 130336650 Svalbard | Svalbard | Holarctic | BR 5040130336650 | B. Godzik & K. Grodzinska | 77.26, 16.34 ^1^ | MF180476 | - | MF180589 |
| BR 026484038 Iceland | Iceland | Holarctic | BR 5040026484038 | C. Van den Berghen | 65.69, -18.12 ^1^ | - | MF180303 | - |
| BR 138015816 Russia, Chukotka | Russia | Holarctic | BR 5040138015816 | O. Dopokuka | 69.76, 162.14 ^1^ | MF180477 | - | - |
| BR 282936845 Japan, Hokkaido | Japan | Holarctic | BR 5040282936845 | s.n. | 43.11, 144.4 ^1^ | MF180478 | - | - |
| BR 120329495 Russia | Russia | Holarctic | BR 5040120329495 | V. Vašák | 55.29, 100.95 ^1^ | - | - | MF180590 |
| AAS 3335A Falklands | Falkland Is. | Antarctic region | AAS | s.n. | 69.27, 20.01 ^1^ | MF180479 | - | - |
| EMB Chile | Chile | Antarctic region | BAS dried samples | E.M. Biersma s.n. | -54.94, -67.63 ^1^ | MF180480 | - | - |
| AAS 5457 Falkland Is. | Falkland Is. | Antarctic region | AAS | R.E. Longton 05457 | -51.68, -57.92 | - | MF180304 | - |
| AAS 249B S. Sandwich Is. | S. Sandwich Is. | Antarctic region | AAS | P. Convey 00249B | -56.67, -28.13 | MF180481 | - | MF180591 |
| AAS 24B S. Sandwich Is. | S. Sandwich Is. | Antarctic region | AAS | P. Convey 00024B | -59.43, -27.08 | MF180482 | - | MF180592 |
| AAS 163A S. Sandwich Is. | S. Sandwich Is. | Antarctic region | AAS | P. Convey 00163A | -57.07, -26.7 | MF180483 | - | MF180593 |
| AAS 126A S. Sandwich Is. | S. Sandwich Is. | Antarctic region | AAS | P. Convey 00126A | -57.07, -26.7 | - | MF180305 | MF180594 |
| AAS 5058 South Georgia | South Georgia | Antarctic region | AAS | R.E. Longton 05058 | -54.22, -36.67 | - | MF180306 | - |
| AAS 5066 South Georgia | South Georgia | Antarctic region | AAS | R.E. Longton 05066 | -54.17, -36.72 | - | MF180307 | - |
| AAS 5047 South Georgia | South Georgia | Antarctic region | AAS | R.E. Longton 05047 | -54.23, -36.63 | MF180484 | - | - |
| S. Shetland Is. Ardley I. 2C | S. Shetland Is. | Antarctic region | BAS / Cambridge U. | J. Royles s.n. | -62.22, -58.93 ^1^ | MF180486 | - | MF180595 |
| S. Shetland Is. Ardley I. 2D | S. Shetland Is. | Antarctic region | BAS / Cambridge U. | J. Royles s.n. | -62.22, -58.93 ^1^ | MF180485 | - | MF180596 |
| S. Shetland Is. Ardley I. 1J | S. Shetland Is. | Antarctic region | BAS / Cambridge U. | J. Royles s.n. | -61.11, -55.14 ^1^ | MF180487 | - | MF180597 |
| S. Shetland Is. Elephant I. 1A (1) | S. Shetland Is. | Antarctic region | BAS / Cambridge U. | J. Royles s.n. | -61.11, -55.14 ^1^ | - | MF180308 | - |
| S. Shetland Is. Elephant I. 1A (2) | S. Shetland Is. | Antarctic region | BAS / Cambridge U. | J. Royles s.n. | -61.11, -55.14 ^1^ | - | MF180309 | - |
| S. Shetland Is. Elephant I. 1A (4) | S. Shetland Is. | Antarctic region | BAS / Cambridge U. | J. Royles s.n. | -61.11, -55.14 ^1^ | - | MF180310 | - |
| S. Shetland Is. Elephant I. 1B (1,2) | S. Shetland Is. | Antarctic region | BAS / Cambridge U. | J. Royles s.n. | -61.11, -55.14 ^1^ | MF180488 | - | MF180598 |
| S. Shetland Is. Elephant I. 1B (3) | S. Shetland Is. | Antarctic region | BAS / Cambridge U. | J. Royles s.n. | -61.11, -55.14 ^1^ | MF180489 | - | - |
| S. Shetland Is. Elephant I. 1B (4,5) | S. Shetland Is. | Antarctic region | BAS / Cambridge U. | J. Royles s.n. | -61.11, -55.14 ^1^ | MF180490 | - | - |
| S. Shetland Is. Elephant I. 1E | S. Shetland Is. | Antarctic region | BAS / Cambridge U. | J. Royles s.n. | -61.11, -55.14 ^1^ | MF180491 | - | MF180599 |
| S. Shetland Is. Elephant I. 1J | S. Shetland Is. | Antarctic region | BAS / Cambridge U. | J. Royles s.n. | -61.11, -55.14 ^1^ | MF180492 | - | MF180600 |
| S. Shetland Is. Elephant I. 2B | S. Shetland Is. | Antarctic region | BAS / Cambridge U. | J. Royles s.n. | -61.11, -55.14 ^1^ | MF180493 | - | MF180601 |
| Antarctic Peninsula, Norsel Point 1H | Antarctic Peninsula | Antarctic region | BAS / Cambridge U. | J. Royles s.n. | -64.45, -64.05 ^1^ | MF180494 | - | - |
| Antarctic Peninsula, Norsel Point 1E | Antarctic Peninsula | Antarctic region | BAS / Cambridge U. | J. Royles s.n. | -64.45, -64.05 ^1^ | MF180495 | - | - |
| AAS 1690 Ant. Pen., Palmer Coast | Antarctic Peninsula | Antarctic region | AAS | D.C. Lindsay 01690 | -63.55, -59.85 | MF180496 | - | - |
| AAS 824 Ant. Pen., Axander I. | Antarctic Peninsula | Antarctic region | AAS | P. Convey 00824 | -69.37, -71.85 | MF180497 | - | MF180602 |
| AAS 832 Ant. Pen., Axander I. | Antarctic Peninsula | Antarctic region | AAS | P. Convey 00832 | -69.37, -71.85 | MF180498 | - | MF180603 |
| AAS 1318 Ant. Pen., Danco Coast | Antarctic Peninsula | Antarctic region | AAS | R.E. Longton 01318 | -64.82, -64.03 | - | MF180311 | - |
| AAS 2 Ant Pen., Trinity Coast | Antarctic Peninsula | Antarctic region | AAS | C.G. Brading 00002 | -63.4, -57 | - | MF180312 | - |
| AAS 4269 Antarctic Peninsula | Antarctic Peninsula | Antarctic region | AAS | R.I.L. Smith 04269 | -65.23, -64.23 | MF180499 | - | MF180604 |
| AAS 4897 Antarctic Peninsula | Antarctic Peninsula | Antarctic region | AAS | R.I.L. Smith 04897 | -65.27, -64.13 | MF180500 | - | MF180605 |
| AAS 27 Ant. Pen., Trinity Coast | Antarctic Peninsula | Antarctic region | AAS | C.G. Brading 00027 | -63.4, -57 | MF180501 | - | MF180606 |
| AAS 3368a Ant. Pen., Graham Coast | Antarctic Peninsula | Antarctic region | AAS | R.I.L. Smith 03368A | -65.32, -64.17 | MF180502 | - | MF180607 |
| AAS 700a Ant. Pen., Palmer Coast | Antarctic Peninsula | Antarctic region | AAS | R.I.L. Smith 00700A | -63.75, -60.67 | MF180503 | - | MF180608 |
| *Polytrichum piliferum* |  |  |  |  |  |  |  |  |
| H 3120679 Canada, Yukon | Canada | Holarctic | H 3120679 | D. Vitt | 64.37, -136.46 | MF180504 | - | - |
| H 3120727 Canada, Quebec | Canada | Holarctic | H 3120727 | D. Weber | 57.06, -65.23 | MF180505 | - | - |
| BR 225742236 Canada, British Columbia | Canada | Holarctic | BR 5040225742236 | F. Demaret | 50.12, -123.03 ^1^ | MF180506 | - | - |
| BR 282869174 Canada, Quebec | Canada | Holarctic | BR 5040282869174 | J.L. De Sloover | 45.8, -74.64 ^1^ | - | MF180313 | - |
| H WS70685 Canada | Canada | Holarctic | H WS70685 | W.D. Schofield | 62.34, -128.32 | - | MF180314 | - |
| H 3120695 Canada, Newfoundland | Canada | Holarctic | H 3120695 | G. Brassard | 48.8, -54.21 ^1^ | - | MF180315 | - |
| H 3120704 Canada, Ontario | Canada | Holarctic | H 3120704 | R.R. Ireland | 45, -81.15 | - | MF180316 | - |
| BR 282809545 Canada, Newfoundland | Canada | Holarctic | BR 5040282809545 | J. Bridgland | 53.7, -57.02 | - | - | MF180609 |
| H 3120547 U.S.A., Missouri | U.S.A. | Holarctic | H 3120547 | B. Allen | 37.65, -90.68 ^1^ | - | MF180317 | - |
| H 3120769 U.S.A., Hawaii | U.S.A. | Holarctic | H 3120769 | W.J. Hoe | 20.71, -156.15 ^1^ | - | MF180318 | - |
| BR 016853721 U.S.A., Alaska | U.S.A. | Holarctic | BR 5040016853721 | M. Lewis | 66.92, -156.92 | - | MF180319 | - |
| BR 282846915 U.S.A., Hawaii | U.S.A. | Holarctic | BR 5040282846915 | W.J. Hoe | 20.64, -156.12 ^1^ | - | MF180320 | - |
| H 3120771 U.S.A., Hawaii | U.S.A. | Holarctic | H 3120771 | W.J. Hoe | 20.71, -156.14 ^1^ | - | MF180321 | - |
| BR 225791722 U.S.A., Washington | U.S.A. | Holarctic | BR 5040225791722 | F.J. Hermann | 46.83, -121.76 ^1^ | MF180507 | - | MF180610 |
| BR 017183148 U.S.A., Minnesota | U.S.A. | Holarctic | BR 5040017183148 | J.A. Janssens | 45.4, -92.66 | - | MF180322 | MF180611 |
| BR 019183740 U.S.A., Washington | U.S.A. | Holarctic | BR 5040019183740 | M.P. Harthill | 47.97, -123.5 ^1^ | - | MF180323 | MF180612 |
| BR 282801464 U.S.A., Missouri | U.S.A. | Holarctic | BR 5040282801464 | B. Allen | 37.92, -93.75 ^1^ | - | MF180324 | MF180613 |
| BR 282833786 U.S.A., Washington | U.S.A. | Holarctic | BR 5040282833786 | M.P. Harthill | 47.97, -123.5 ^1^ | - | MF180325 | MF180614 |
| H 3120730 Greenland | Greenland | Holarctic | H 3120730 | K. Holmen | 66.06, -37.13 | MF180508 | - | - |
| TUR WW1152 Poland | Poland | Holarctic | TUR WW1152 | W. Wilczynska | 51.03, 17.15 ^1^ | MF180509 | - | - |
| BR 016108067 Portugal | Portugal | Holarctic | BR 5040016108067 | Stud. Biol. Rheno-trai in Itinere | 41.68, -8.43 ^1^ | MF180510 | - | - |
| BR 189174241 Georgia | Georgia | Holarctic | BR 5040189174241 | V. Vašák | 41.71, 44.91 ^1^ | MF180511 | - | - |
| BR 225821054 United Kingdom | United Kingdom | Holarctic | BR 5040225821054 | G. Raeymaekers | 50.59, -3.95 ^1^ | MF180512 | - | - |
| BR 341607709 Norway | Norway | Holarctic | BR 5040341607709 | T. Arts | 61.33, 8.15 ^1^ | MF180513 | - | - |
| BR 117747868 France | France | Holarctic | BR 5040117747868 | J.-P. Frahm | 47.85, 6.59 ^1^ | MF180514 | - | MF180615 |
| BR 217565911 Slovakia | Slovakia | Holarctic | BR 5040217565911 | J. Bouharmont | 48.93, 19.75 ^1^ | MF180515 | - | MF180616 |
| BR 282836817 France | France | Holarctic | BR 5040282836817 | J.L. De Sloover | 45.01, 6.12 ^1^ | MF180516 | - | MF180617 |
| AY396439 Finland | Finland | Holarctic | H 6205 | J. Hyvönen | 60.3, 24.28 ^1^ | - | AY396439 | - |
| H 3120607 Germany | Germany | Holarctic | H 3120607 | H. Hämäläinen | 59.27, 13.02 | - | MF180326 | - |
| H 3120660 Canary Is. | Spain | Holarctic | H 3120660 | R. Rajalin | 28.36, -17.5 | - | MF180327 | - |
| BR 031803848 Switzerland | Switzerland | Holarctic | BR 5040031803848 | A. Lawalrée | 46.15, 7.06 ^1^ | - | MF180328 | - |
| H B3992 Faroe Is. | Faroe Is. | Holarctic | H B3992 | s.n. | 62.06, -6.83 ^1^ | - | MF180329 | - |
| H 6205 Finland | Finland | Holarctic | H 6205 | J. Hyvönen | 60.3, 24.28 ^1^ | - | MF180330 | - |
| H 3120658 Madeira | Portugal | Holarctic | H 3120658 | R. Ruotsalo-Aario & L. Aario | 32.73, -17.05 ^1^ | - | MF180331 | - |
| TUR 88-130 Finland | Finland | Holarctic | TUR 88-130 | Y. Mäkinen | 60.63, 21.29 ^1^ | - | MF180332 | - |
| BR 018305693 Poland | Poland | Holarctic | BR 5040018305693 | S. Lisowski | 49.2, 19.75 ^1^ | - | MF180333 | - |
| BR 022118029 Sweden | Sweden | Holarctic | BR 5040022118029 | H. Möller | 58.57, 11.37 ^1^ | - | MF180334 | - |
| BR 022119033 Sweden | Sweden | Holarctic | BR 5040022119033 | H. Möller | 61.29, 13.91 ^1^ | - | MF180335 | - |
| BR 026466829 Sweden | Sweden | Holarctic | BR 5040026466829 | L. Cooreman | 68.35, 18.83 ^1^ | - | MF180336 | - |
| AAS 157 Falkland Is. | Falkland Is. | Holarctic | AAS | J.J. Engel 00157 | -51.75, -59.5 | - | MF180337 | - |
| BR 225749303 Portugal | Portugal | Holarctic | BR 5040225749303 | Stud. Biol. Rheno-trai in Itinere | 40.45, -7.64 ^1^ | - | MF180338 | - |
| BR 282891397 Norway | Norway | Holarctic | BR 5040282891397 | Stud. Biol. Rheno-Trai in Itinere | 62.15, 9.17 ^1^ | - | MF180339 | - |
| BR 314397200 Finland | Finland | Holarctic | BR 5040314397200 | T. Arts | 69.05, 20.82 ^1^ | - | MF180340 | - |
| BR 341596591 Ireland | Ireland | Holarctic | BR 5040341596591 | T. Arts | 53.4, -9.96 ^1^ | - | MF180341 | - |
| BR 103358535 France | France | Holarctic | BR 5040103358535 | J.L. De Sloover | 43.42, 2.46 ^1^ | - | MF180342 | MF180618 |
| BR 207875044 Switzerland | Switzerland | Holarctic | BR 5040207875044 | J.A.E. Slembrouck | 47.1, 9.61 ^1^ | - | MF180343 | MF180619 |
| BR 225751320 Poland | Poland | Holarctic | BR 5040225751320 | J. Mickiewicz | 52.33, 20.44 ^1^ | - | MF180344 | MF180620 |
| BR 251012730 Italy | Italy | Holarctic | BR 5040251012730 | M. Onraedt | 45.68, 6.88 ^1^ | - | MF180345 | MF180621 |
| BR 282803482 Faroe Is. | Faroe Is. | Holarctic | BR 5040282803482 | J. Lewinsky | 62.3, -6.72 | - | MF180346 | MF180622 |
| BR 089481470 Poland | Poland | Holarctic | BR 5040089481470 | S. Lisowski, A. Rusinska, & I. Melosik | 50.83, 23.4 | - | - | MF180623 |
| H 3120647 Russia | Russia | Holarctic | H 3120647 | P. Alanko | 51.34, 139.51 | MF180517 | - | - |
| AAS 287 South Georgia | South Georgia | Holarctic | AAS | G.C.S. Clarke 00287 | -54.28, -36.5 | MF180518 | - | MF180624 |
| H 3120653 China, Jilin | China | Holarctic | H 3120653 | T. Koponen | 41.3, 128.1 | - | MF180347 | - |
| BR 189173237 Georgia | Georgia | Holarctic | BR 5040189173237 | V. Vašák | 42.93, 41.11 ^1^ | - | MF180348 | - |
| BR 119154381 Georgia | Georgia | Holarctic | BR 5040119154381 | V. Vašák | 42.93, 41.11 ^1^ | - | MF180349 | MF180625 |
| BR 120327477 Georgia | Georgia | Holarctic | BR 5040120327477 | V. Vašák | 43, 41.02 ^1^ | - | MF180350 | MF180626 |
| BOL 109447 South Africa | South Africa | Holarctic | BOL 109447 | T.A. Hedderson | -32.36, 19.12 | - | MF180351 | - |
| BOL 109448 South Africa | South Africa | Holarctic | BOL 109448 | T.A. Hedderson | -32.23, 19.1 | - | MF180352 | - |
| H JH5997 Chile | Chile | Antarctic region | H JH5997 | J. Hyvönen | -38.42, -71.41 | MF180519 | - | - |
| H 3120779 Argentina | Argentina | Antarctic region | H 3120779 | A. Kalela | -40.66, -71.42 ^1^ | - | MF180353 | - |
| H BR23-2-66 Chile | Chile | Antarctic region | H BR23-2-66 | B. Ruthsatz | -39.93, -72.03 ^1^ | - | MF180354 | - |
| H 2168 Argentina | Argentina | Antarctic region | H 2168 | J. Hyvönen | -53.24, -68.5 | - | MF180355 | - |
| H 2282 Argentina | Argentina | Antarctic region | H 2282 | J. Hyvönen | -53.46, -69.47 | - | MF180356 | - |
| H 4970 Argentina | Argentina | Antarctic region | H 4970 | J. Hyvönen | -50.1, -72.47 | - | MF180357 | - |
| H 3120781 Falkland Is. | Falkland Is. | Antarctic region | H 3120781 | J. Engel | -51.21, -60.42 | - | MF180358 | - |
| H RLS73 South Georgia | South Georgia | Antarctic region | H RLS73 | R.I.L. Smith | -54.23, -36.58 ^3^ | MF180520 | - | - |
| H RLS75 South Georgia | South Georgia | Antarctic region | H RLS75 | R.I.L. Smith | -54.1, -36.42 | MF180521 | - | - |
| H 3120767 South Georgia | South Georgia | Antarctic region | H 3120767 | R. Longton | -54.28, -36.5 ^1^ | - | MF180359 | - |
| AAS 67 S. Shetland Is. | S. Shetland Is. | Antarctic region | AAS | R.I.L. Smith 00067 | -62.98, -60.58 | - | MF180360 | - |
| S. Shetland Is. Barrientos I. 3C | S. Shetland Is. | Antarctic region | BAS / Cambridge U. | J. Royles s.n. | -62.41, -59.75 ^1^ | MF180522 | - | - |
| H 3120778 S. Shetland Is. | S. Shetland Is. | Antarctic region | H 3120778 | R. Ochyra | -62.04, -58.25 | - | MF180361 | - |
| AAS 3332 Antarctic Peninsula | Antarctic Peninsula | Antarctic region | AAS | R.I.L. Smith 3332 | -65.42, -64.23 | - | MF180362 | - |
| AAS 2770A Antarctic Peninsula | Antarctic Peninsula | Antarctic region | AAS | M. Sharp 02770A | -67.62, -64.75 | MF180523 | - | - |
| *Polytrichastrum alpinum* |  |  |  |  |  |  |  |  |
| TUR 63-18-11, U.S.A., Colorado | U.S.A. | Holarctic | TUR 63-18-11 | A. Vaarama | 39.6, -105.64 | MF180524 | - | - |
| BR 282719615 U.S.A., Montana | U.S.A. | Holarctic | BR 5040282719615 | L. E. Anderson | 48.17, -115.83 ^1^ | MF180525 | - | MF180627 |
| H 3118718 Canada, Ontario | Canada | Holarctic | H 3118718 | R.R. Ireland | 45.04, -79.45 | - | MF180363 | - |
| H 3118657 USA, Minnesota | USA | Holarctic | H 3118657 | B. Allen | 47.79, -90.19 | - | MF180364 | - |
| H 3118596 Canada, Newfoundland | Canada | Holarctic | H 3118596 | T.A. Hedderson | 48.35, -53.55 | - | MF180365 | - |
| L SN20 Svalbard | Svalbard | Holarctic | L SN20 | E.M. Biersma & S. Lubbe | 78.08, 18.55 | MF180526 | - | - |
| BR 282697395 Greenland | Greenland | Holarctic | BR 5040282697395 | J. Lewinsky | 63.93, -50.92 | MF180527 | - | - |
| BR 138177491 Switzerland | Switzerland | Holarctic | BR 5040138177491 | J. Bouharmont | 46.52, 9.9 ^1^ | MF180528 | - | - |
| H 10.7.85 Czech Republic | Czech Republic | Holarctic | H 10.7.85 | J. Vána | 50.68, 15.65 | - | MF180366 | - |
| H 3118412 Faroe Is. | Faroe Is. | Holarctic | H 3118412 | J. Lewinsky | 62.02, -7.13 | - | MF180367 | - |
| H JP10813 Finland | Finland | Holarctic | H JP10813 | J. Pykälä | 60.13, 23.49 | - | MF180368 | - |
| H 3118511 Russia, Perm Prov. | Russia | Holarctic | H 3118511 | M. Ignatov & A. Bezgodov | 58.48, 58.28 | - | MF180369 | - |
| H SH102 Slovakia | Slovakia | Holarctic | H SH102 | S. Huttunen & H. Jalkanen | 49.11, 19.59 | - | MF180370 | - |
| H 3118486 Poland | Poland | Holarctic | H 3118486 | H. Bednarek-Ochyra & R. Ochyra | 49.24, 19.92 ^1^ | - | MF180371 | - |
| H SH1083 Russia | Russia | Holarctic | H SH1083 | S. Huttunen | 67.09, 31.51 | - | MF180372 | - |
| H SH928 Russia | Russia | Holarctic | H SH928 | S. Huttunen & H. Wahlberg | 61.45, 30.47 | - | MF180373 | - |
| TROM B 320018 Norway | Norway | Holarctic | TROM B 320018 | I. Hagen | 63.42, 10.45 ^1^ | - | MF180374 | - |
| BR 138106750 Austria | Austria | Holarctic | BR 5040138106750 | J. Bouharmont | 47.19, 13.3 ^1^ | - | - | MF180628 |
| BR 120322427 Georgia | Georgia | Holarctic | BR 5040120322427 | V. Vašák | 42.4, 43.94 ^1^ | MF180529 | - | - |
| H 3118618 China, Xinjiang | China | Holarctic | H 3118618 | B. Tan | 43.32, 89.44 | - | MF180375 | - |
| H JH3524 Taiwan | Taiwan | Holarctic | H JH3524 | J. Hyvönen | 24.08, 121.17 | - | MF180376 | - |
| H JH3538 Taiwan | Taiwan | Holarctic | H JH3538 | J. Hyvönen | 24.08, 121.17 | - | MF180377 | - |
| H JH3709 Taiwan | Taiwan | Holarctic | H JH3709 | J. Hyvönen | 23.27, 120.57 | - | MF180378 | - |
| H MI97543 Russia | Russia | Holarctic | H MI97543 | M. Ignatov | 51.32, 133.54 | - | MF180379 | - |
| H 3118602 China | China | Holarctic | H 3118602 | T. Koponen | 41.3, 128.1 | - | MF180380 | - |
| H 3118745 Australia | Australia | Australasia | H 3118745 | H. Streimann | -36.53, 147.18 | - | MF180381 | - |
| Macquarie I. | Macquarie I. | Australasia | BAS frozen samples | D. Hodgson s.n. | -54.62, 158.86 ^1^ | MF180530 | - | - |
| H JH6872 Argentina | Argentina | Antarctic region | H JH6872 | J. Hyvönen | -54.49, -68.33 | - | MF180382 | - |
| AAS 5456 Falkland Is. | Falkland Is. | Antarctic region | AAS | R.E. Longton 05456 | -51.67, -57.92 | - | MF180383 | - |
| H RLS67 S. Orkney Isl. | S. Orkney Is. | Antarctic region | H RLS67 | R.I.L. Smith | -60.72, -45.6 ^3^ | - | MF180384 | - |
| H RLS71 South Georgia | South Georgia | Antarctic region | H RLS71 | R.I.L. Smith | -54.16, -36.3 | - | MF180385 | - |
| H RLS76 South Georgia | South Georgia | Antarctic region | H RLS76 | R.I.L. Smith | -54.1, -36.42 | - | MF180386 | - |
| S. Shetland Is., Deception I. | S. Shetland Is. | Antarctic region | BAS frozen samples | E.M. Biersma s.n. | -62.98, -60.71 ^1^ | MF180531 | - | - |
| S. Shetland Is., Arctowski | S. Shetland Is. | Antarctic region | BAS frozen samples | E.M. Biersma s.n. | -62.16, -58.47 ^1^ | MF180532 | - | - |
| S. Shetland Is. Hannes Point | S. Shetland Is. | Antarctic region | BAS frozen samples | E.M. Biersma s.n. | -62.64, -60.6 ^1^ | MF180533 | - | - |
| S. Shetland Is. Barrientos I. 1A | S. Shetland Is. | Antarctic region | BAS / Cambridge U. | J. Royles s.n. | -62.41, -59.75 ^1^ | MF180534 | - | - |
| S. Shetland Is. Barrientos I. 1B | S. Shetland Is. | Antarctic region | BAS / Cambridge U. | J. Royles s.n. | -62.41, -59.75 ^1^ | MF180535 | - | MF180629 |
| S. Shetland Is. Barrientos I. 1C (1,2,4) | S. Shetland Is. | Antarctic region | BAS / Cambridge U. | J. Royles s.n. | -62.41, -59.75 ^1^ | - | MF180387 | MF180630 |
| S. Shetland Is. Barrientos I. 1C (3) | S. Shetland Is. | Antarctic region | BAS / Cambridge U. | J. Royles s.n. | -62.41, -59.75 ^1^ | MF180536 | - | MF180631 |
| S. Shetland Is. Barrientos I. 1C (5) | S. Shetland Is. | Antarctic region | BAS / Cambridge U. | J. Royles s.n. | -62.41, -59.75 ^1^ | - | MF180388 | - |
| S. Shetland Is. Barrientos I. 1D | S. Shetland Is. | Antarctic region | BAS / Cambridge U. | J. Royles s.n. | -62.41, -59.75 ^1^ | - | MF180389 | MF180632 |
| S. Shetland Is. Barrientos I. 1D (2) | S. Shetland Is. | Antarctic region | BAS / Cambridge U. | J. Royles s.n. | -62.41, -59.75 ^1^ | - | MF180390 | - |
| S. Shetland Is. Barrientos I. 1D (5) | S. Shetland Is. | Antarctic region | BAS / Cambridge U. | J. Royles s.n. | -62.41, -59.75 ^1^ | - | MF180391 | - |
| S. Shetland Is. Barrientos I. 1E | S. Shetland Is. | Antarctic region | BAS / Cambridge U. | J. Royles s.n. | -62.41, -59.75 ^1^ | MF180537 | - | - |
| S. Shetland Is. Barrientos I. 3A | S. Shetland Is. | Antarctic region | BAS / Cambridge U. | J. Royles s.n. | -62.41, -59.75 ^1^ | MF180538 | - | - |
| H 12.II.80 S. Shetland Is. | S. Shetland Is. | Antarctic region | H 12.II.80 | Y. Petrov | -62, -58 ^1^ | - | MF180392 | - |
| AAS 115 Ant. Pen., Fallieres Coast | Antarctic Peninsula | Antarctic region | AAS | J. Killingbeck 00115 | -67.73, -68.42 | - | MF180393 | - |
| H RLS83 Ant. Pen., Lagoon I. | Antarctic Peninsula | Antarctic region | H RLS83 | R.I.L. Smith | -67.58, -68.25 ^3^ | - | MF180394 | - |
| AAS 2304 Ant. Pen., Danco Coast | Antarctic Peninsula | Antarctic region | AAS | R.E. Longton 02304 | -64.58, -63.5 | - | MF180395 | - |
| AAS 25 Ant. Pen., Trinity Coast | Antarctic Peninsula | Antarctic region | AAS | C.G. Brading 00025 | -63.42, -57.02 | - | MF180396 | - |
| AAS 427 Ant. Pen., Fallieres Coast | Antarctic Peninsula | Antarctic region | AAS | B.J. Taylor 00427 | -67.73, -68.42 | - | MF180397 | - |
| AAS 686B Ant. Pen., Graham Coast | Antarctic Peninsula | Antarctic region | AAS | R.W.M. Corner 00686B | -65.27, -64.13 | - | MF180398 | - |
| Longitudes and latitudes not provided with sample. Approximate location found via:  ^1^ = from http://mynasadata.larc.nasa.gov/latitudelongitude-finder/, ^2^ = from http://tools.wmflabs.org/geohack/, ^3^ = coordinates from similar location from AAS herbarium website | | | | | | | | |


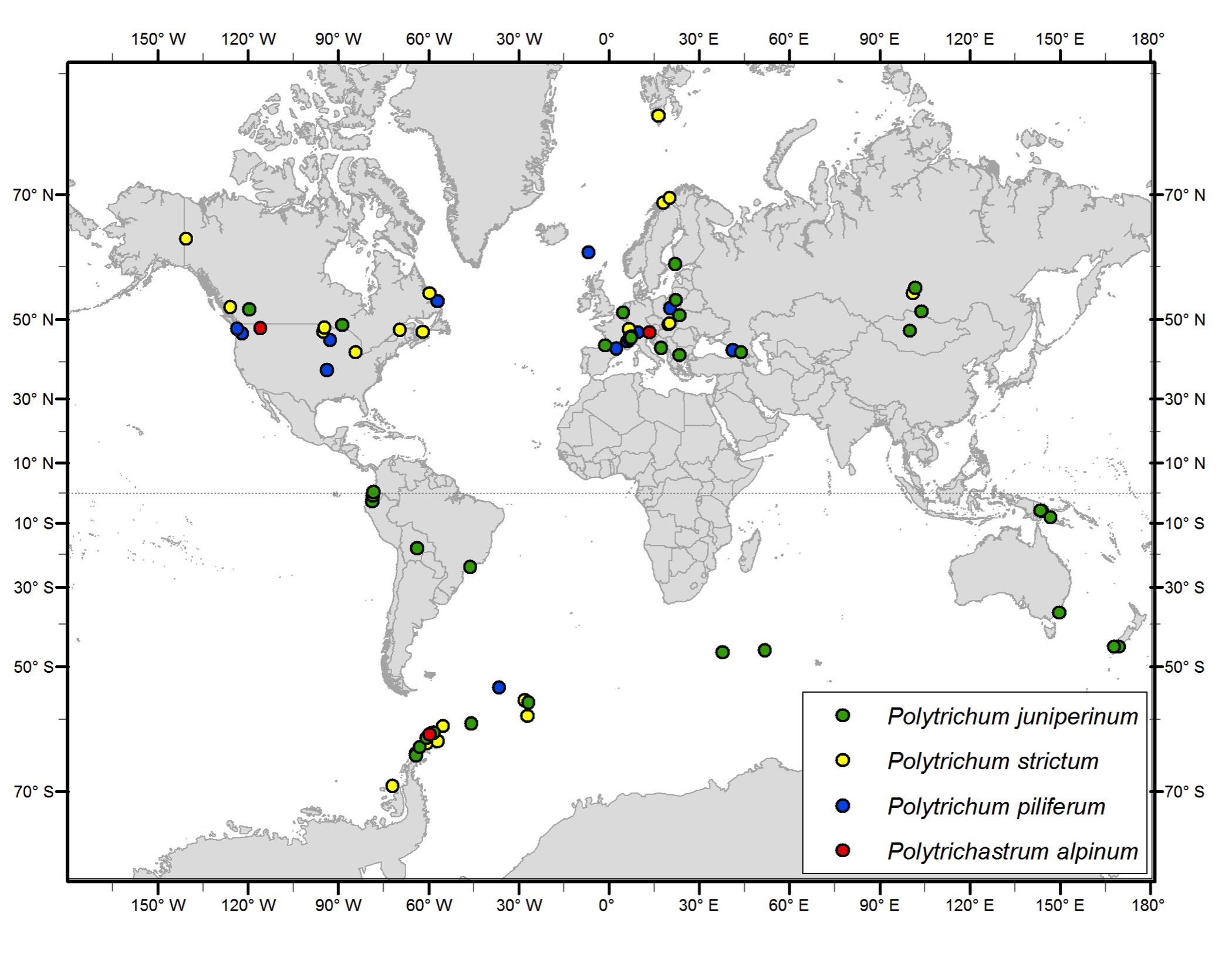


**Fig. S1**. Locations of *trnL-F* samples of *Polytrichum juniperinum* (green), *Polytrichum strictum* (yellow), *Polytrichum piliferum* (blue) and *Polytrichastrum alpinum* (red).


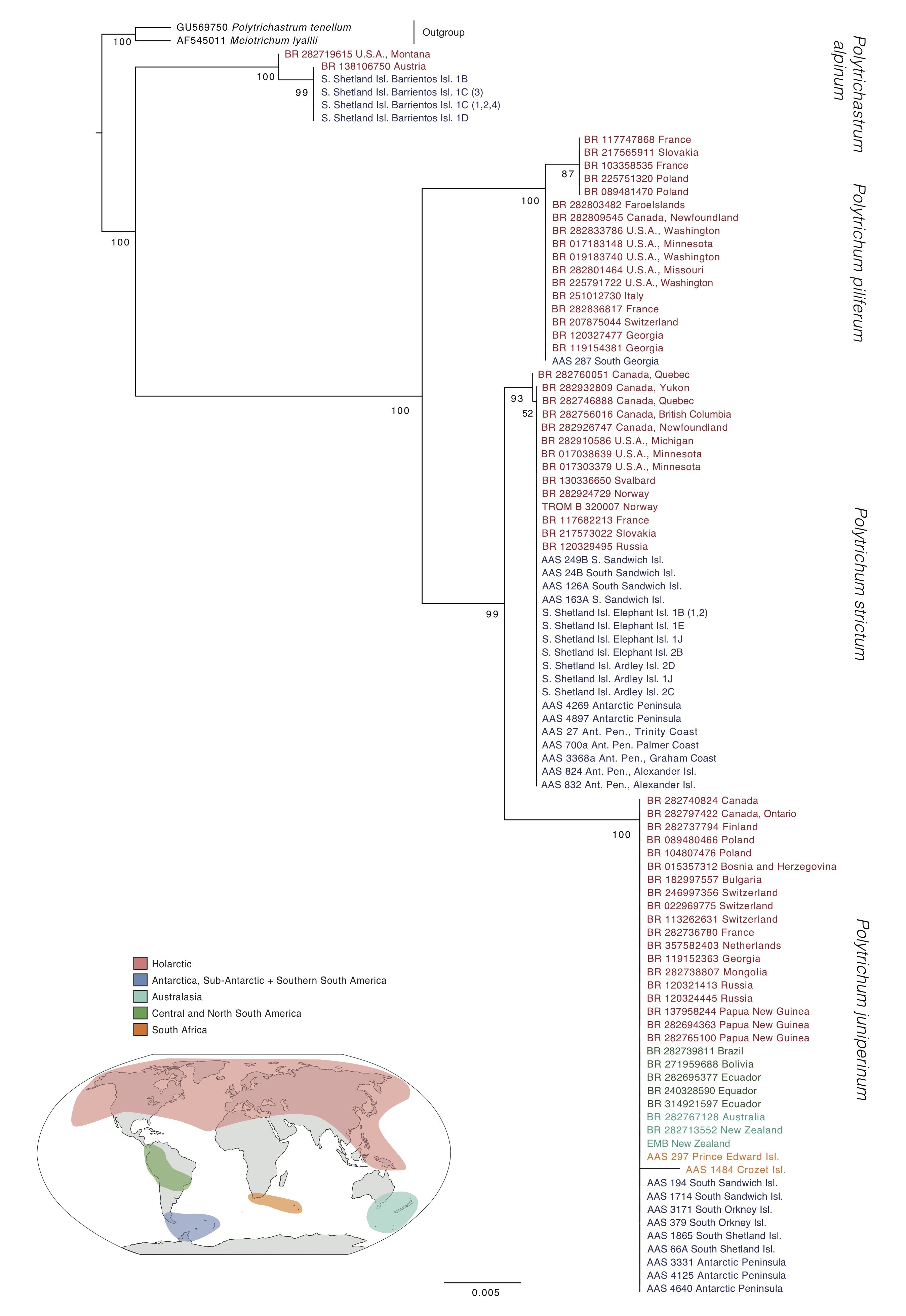


**Fig. S2.** Maximum Likelihood tree of *trnL-F* for *Polytrichastrum alpinum,* *Polytrichum. piliferum*, *P. strictum* and *P. juniperinum*. Bootstrap support is provided below or next to the relevant node. Taxon colours refer to the different continents or regions (see map). Outgroups are indicated in black. The scale bar represents the mean number of nucleotide substitutions per site.

**
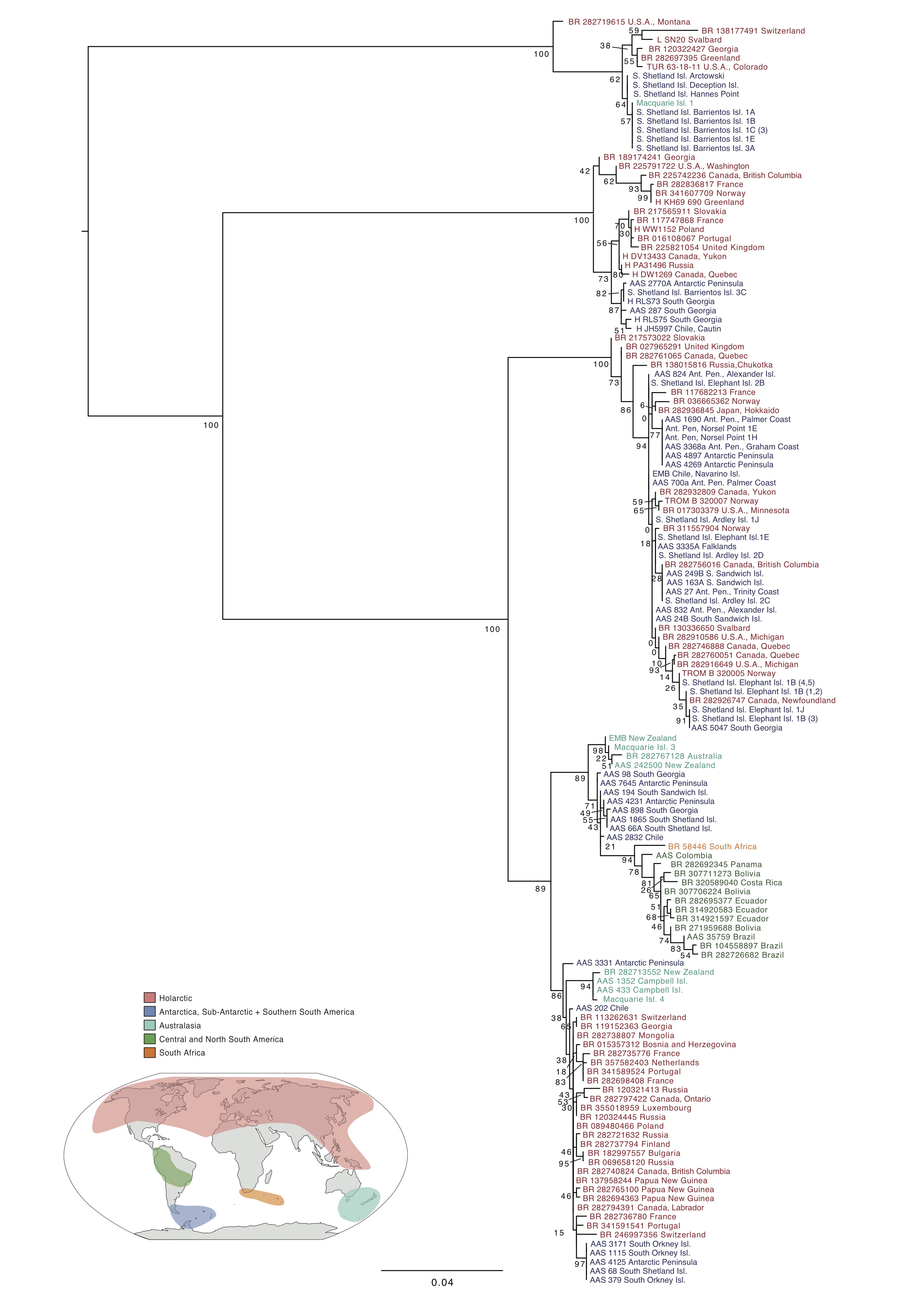
**

**Fig. S3.** Maximum Likelihood tree of *ITS* for *Polytrichastrum alpinum,* *Polytrichum. piliferum*, *P. strictum* and *P. juniperinum*. Bootstrap support is provided below or next to the relevant node. Taxon colours refer to the different continents or regions (see map). The scale bar represents the mean number of nucleotide substitutions per site.

|  |
| --- |

| **Table S2.** Genetic diversity indices, demographic and spatial expansion model test and neutrality tests (Tajima’s D and Fu’s Fs) for *ITS* 1+2 within the species *Polytrichum juniperinum*, *P. strictum*, *P. piliferum* and *Polytrichastrum alpinum*. Parameters were only calculated for populations with *n*>10. The different ABGD-inferred clusters PJ1-4 of *P. juniperinum* are shown in Fig. 2. None of the SSD or HRI p-values were significant. | | | | | | | | | | | | | | |
| --- | --- | --- | --- | --- | --- | --- | --- | --- | --- | --- | --- | --- | --- | --- |
|  |  |  |  | |  |  | Demographic expansion | | Spatial expansion | | Neutrality tests: | |  | |
| Clade | *n* | bp^a^ | π | S | *h* | | SSD | HRI | SSD | HRI | Tajima's D (*P*) | Fu's Fs (*P*) | | |
| *Polytrichastrum alpinum* (overall) | 15 | 782 | 0.010 ± 0.005 | 33 | 0.876 ± 0.070 | | 0.062 | 0.080 | 0.052 | 0.080 | -1.574 (0.047*) | 0.115 (0.529) | | |
| *Polytrichum piliferum* (overall) | 20 | 1304 | 0.017 ± 0.009 | 46 | 0.990 ± 0.019 | | 0.013 | 0.013 | 0.014 | 0.013 | 0.177 (0.622) | -3.062 (0.093) | | |
| *Polytrichum strictum* (overall) | 44 | 979 | 0.006 ± 0.003 | 30 | 0.986 ± 0.009 | | 0.001 | 0.005 | 0.001 | 0.005 | -0.846 (0.217) | -2.915 (0.153) | | |
| *Polytrichum juniperinum* (overall) | 61 | 993 | 0.035 ± 0.017 | 88 | 0.996 ± 0.005 | | 0.007 | 0.002 | 0.007 | 0.002 | 0.263 (0.682) | -13.365 (0.006*) | |  |
| “SH Clade” (PJ1+PJ2+PJ3) | 25 | 994 | 0.024 ± 0.012 | 54 | 0.997 ± 0.013 | | 0.006 | 0.008 | 0.011 | 0.008 | -0.336 (0.694) | -7.060 (0.011*) | | |
| S. Africa, N./Centr. S. America (PJ3) | 13 | 896 | 0.012 ± 0.007 | 34 | 1.000 ± 0.030 | | 0.006 | 0.013 | 0.007 | 0.013 | -1.005 (0.156) | -5.444 (0.009*) | | |
| “Bi-hemisphere Clade” (PJ4) | 36 | 961 | 0.011 ± 0.006 | 40 | 0.991 ± 0.011 | | 0.002 | 0.004 | 0.003 | 0.004 | -1.351 (0.069) | -11.525 (0.002*) | | |
| Holarctic + recent Antarctic dispersal event | 30 | 963 | 0.009 ± 0.005 | 26 | 0.986 ± 0.016 | | 0.002 | 0.005 | 0.003 | 0.005 | -1.100 (0.133) | -9.031 (0.003*) | | |
| *n*: no. of sequences; bp^a^: no. of usable base pairs (loci <5.0% missing data); π: nucleotide diversity (average over locus); S: No. of sites with substitutions; *h*: gene diversity; SSD: Sum of Squared Deviations; HRI: Harpending's Raggedness Index. For Tajima’s D and Fu’s Fs a *P*<0.05 is significant (*). | | | | | | | | | | | | | | |

**Dating Analyses**:

For all dating analyses we used BEAST v2.2.1 [1], and Tracer v1.6 [2] to examine stationarity and effective sampling (ESS>100) from the posterior distribution. To investigate the divergence times of the different species and populations in the *ITS* dataset, we used the following different calibration approaches:

(I) Two-step analyses:

(I1) In the first step of the two-step age estimations, we assessed the age of the split between *P. piliferum*/(*P. juniperinum* + *P. strictum*), using a larger dataset, which spans all major clades of the Polytrichales and six gene compartments: *rbcL*, *trnL-F, rps4*, *rps4-trnS* (plastid) and *nad5* (mitochondrial) [3]. All BEAST settings followed [3], excepting the addition of *P. strictum* to all gene regions of the dataset, so that all four study-species were represented. We used the same priors as [3], which included 1) a normal (230.53, stdev 22) prior on the age of the MRCA of the Polytrichales [4] (prior 1 in Fig. S4); 2) a uniform prior (37.0, 500) on the *Psilopilum*/*Steereobryon*/*Atrichum*/*Delongia* clade [5] (prior 3 in Fig. S4); and 3) a uniform prior (83.64, 500) on the stem lineage of *Polytrichum* sect. *Polytrichum + P.* sect. *Juniperifolia* (prior 2 in Fig. S4) based on the fossil *Eopolytrichum antiquum* Konopka & al. [3, 6, 7]. Following the most recent palynological analyses of the source material of *E. antiquum* [8-10], we used a lower bound of 83.64 Mya (instead of 80 Mya as in [3]).

Similar to [3], as the precise taxonomic placement of *E. antiquum* is not fully resolved [3, 6, 7], we performed two analyses: one with (I1a) and one without (I1b) *E. antiquum.* Runs (I1a) and (I1b) were run for 1.2×10^9^ and 2.0×10^9^ generations, respectively, sampling every 1.0×10^4^ generations, with a burn-in of 40% and 10%, respectively. Fig. S4 shows the outcome of the (I1a) run with all priors and the key node of interest (*P. piliferum*/(*P. juniperinum* + *P. strictum*)).

(I2) As a second step in the two-step dating analyses we applied the resulting divergence times and corresponding 95% quantiles of the split between *P. piliferum*/(*P. juniperinum* + *P. strictum*) (as calculated from I1), as a secondary prior on the same node in the *ITS* 1+2 dataset (I2). This was done for both analyses with and without *E. antiquum* (in analyses I2a and I2b, respectively).

(II) Molecular rate analysis

We also performed a dating analysis (II) based on a defined *ITS* substitution rate (1.35×10^-3^ subst./site/my) previously applied in bryophytes [11, 12], but originally derived from angiosperms [13, and references therein].

All BEAST analyses based on the *ITS* dataset (Models I2a, I2b and II) had the same settings, except for the particularities of comparing a rate vs. a fossil-based analysis: therefore, we used a clock rate of 1.35×10^-3^ subst./site/my in method II, whereas we used a normal prior on the *P. piliferum*/(*P. juniperinum* + *P. strictum*) node in method I2a (Mean: 35.9185, Sigma: 13.0) and I2b (Mean: 14.1698, Sigma: 4.3). In all runs we used a Strict clock (upper and lower limit of 0.01 and 0.0, respectively), a GTR+G site model and a coalescent Bayesian Skyline tree prior. All MCMC chains were run for 1.0×10^7^ generations, sampling every 1.0×10^3^ generations. Maximum clade credibility trees were constructed in TreeAnnotator v1.8.2 [14], with 10% burn-in removed.


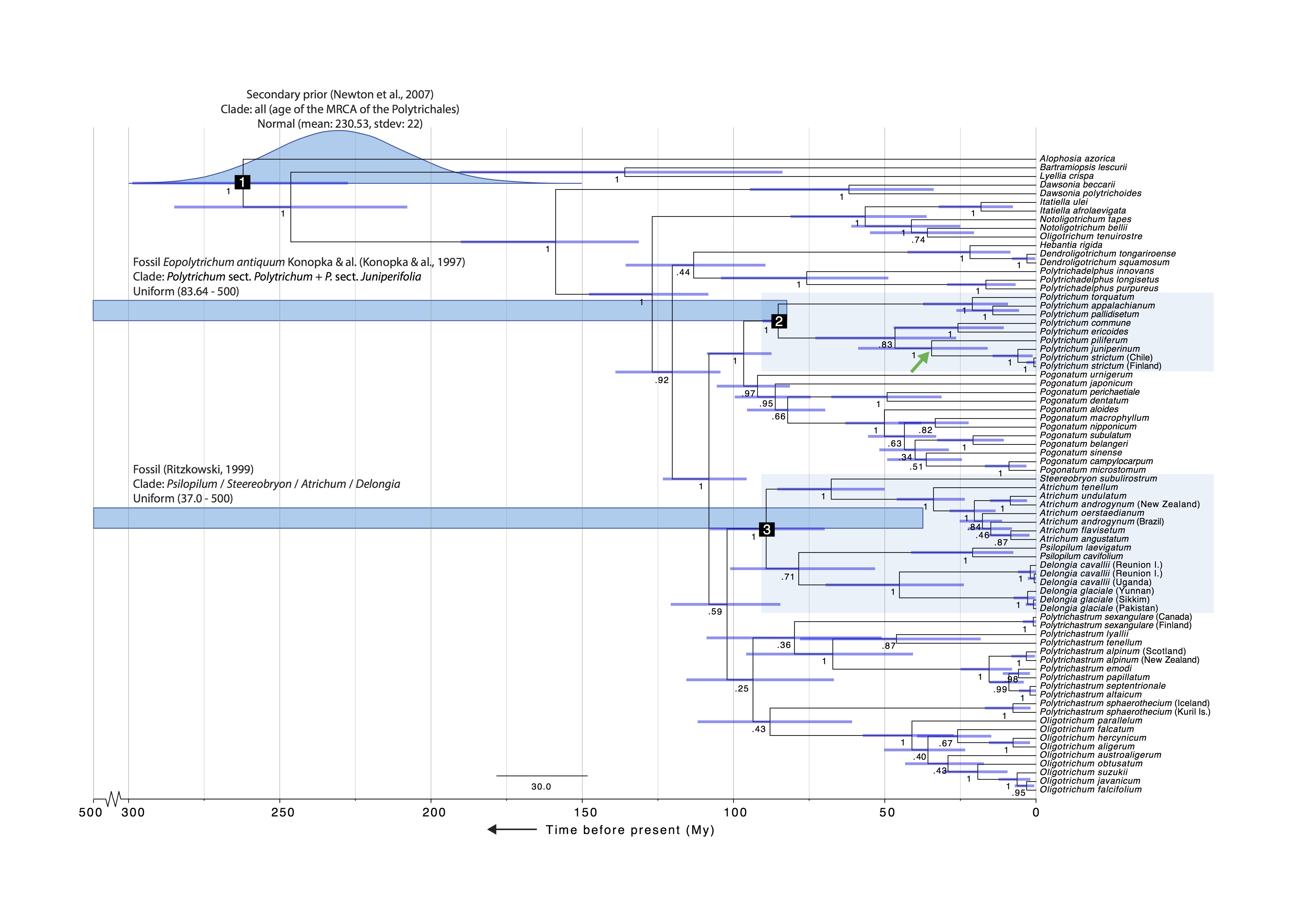


**Fig. S4.** The (I1a) run (including *Eopolytrichum antiquum*), with a graphic representation of all prior information and the key node of interest (*P. piliferum*/(*P. juniperinum* + *P. strictum*); green arrow). The priors include 1) a normal (230.53, stdev 22) prior on the age of the MRCA of the Polytrichales [4]; 2) a uniform prior (83.64, 500) on the stem lineage of *Polytrichum* sect. *Polytrichum + P.* sect. *Juniperifolia*; and 3) a uniform prior (37.0, 500) on the *Psilopilum*/*Steereobryon*/*Atrichum*/*Delongia* clade [5]. See text on two-step dating analyses for more information.

| **Table S3.** Mean ages (95% HDP lower - upper) (my) for the different study species and key clades within *Polytrichum juniperinum* as calculated in BEAST. Dating methods are based on two 2-step dating analyses (Method I), with (I2a) or without (12b) including the taxonomically uncertain fossil *Eopolytrichum antiquum* as a constraint, as well as applying a previously defined *ITS* rate (Method II). The different ABGD-inferred clusters PJ1-4 of *P. juniperinum* are shown in Fig. 2. TMRCA: Time to the Most Recent Common Ancestor; SH: Southern Hemisphere; NH: Northern Hemisphere. The mean clock rate in subst./site/my (95% HDP intervals) is provided for Method (12a) and (12b). | | | |
| --- | --- | --- | --- |
|  | **Method I (two-step)**  *Eopolytrichum antiquum* used as constraint: | | **Method II (rate)** |
| Lineage TMRCA | (I2a) Yes | (I2b) No | Rate Analysis |
| *Polytrichastrum alpinum + Polytrichum spp.* | 45.04 (16.07-76.52) | 22.58 (14.56-31.76) | 130.29 (98.51-164.53) |
| *P. piliferum* *+ P. strictum*/*P. juniperinum* | 32.71 (12.02-53.77) | 16.23 (11.25-22.20) | 94.98 (73.66-117.63) |
| *P. strictum + P. juniperinum* | 9.56 (3.37-16.60) | 4.81 (3.03-6.83) | 27.38 (20.91-34.60) |
| *P. alpinum* | 5.00 (1.64-9.19) | 2.51 (1.35-3.87) | 14.24 (8.92-19.78) |
| SH Clade | 0.47 (0.09-1.05) | 0.24 (0.05-0.47) | 1.38 (0.35-2.62) |
| *P. piliferum* | 2.53 (0.84-4.08) | 1.27 (0.74-1.92) | 7.26 (5.12-9.73) |
| SH Clade | 0.55 (0.15-1.07) | 0.28 (0.10-0.47) | 1.58 (0.71-2.48) |
| *P. strictum* | 2.09 (0.63-3.79) | 1.06 (0.59-1.64) | 5.85 (3.84-8.40) |
| *P. juniperinum* | 4.75 (1.69-8.44) | 2.40 (1.46-3.47) | 13.59 (9.83-17.55) |
| SH Clade – Australasia (PJ1) | 0.51 (0.08-1.10) | 0.27 (0.07-0.51) | 1.53 (0.52-2.77) |
| SH Clade – Antarctic, Sub-Antarctic, S. S. America (PJ2) | 2.27 (0.78-4.06) | 1.16 (0.68-1.71) | 6.54 (4.63-8.52) |
| SH Clade – S. Africa, N. and Centr. S. America (PJ3) | 1.37 (0.48-2.46) | 0.70 (0.40-1.06) | 3.96 (2.71-5.32) |
| NH + SH Clade (PJ4) | 2.11 (0.64-3.88) | 1.05 (0.55-1.63) | 6.01 (3.62-8.46) |
| Recent dispersal Holarctic 🡪 Antarctic | 0.16 (0.01-0.37) | 0.08 (0.01-0.17) | 0.45 (0.06-0.90) |
| Mean clock rate | 4.47×10^-3^ (1.76×10^-3^ - 8.34×10^-3^) | 7.83×10^-3^ (5.52×10^-3^ - 1.00×10^-2^) | N.A. |


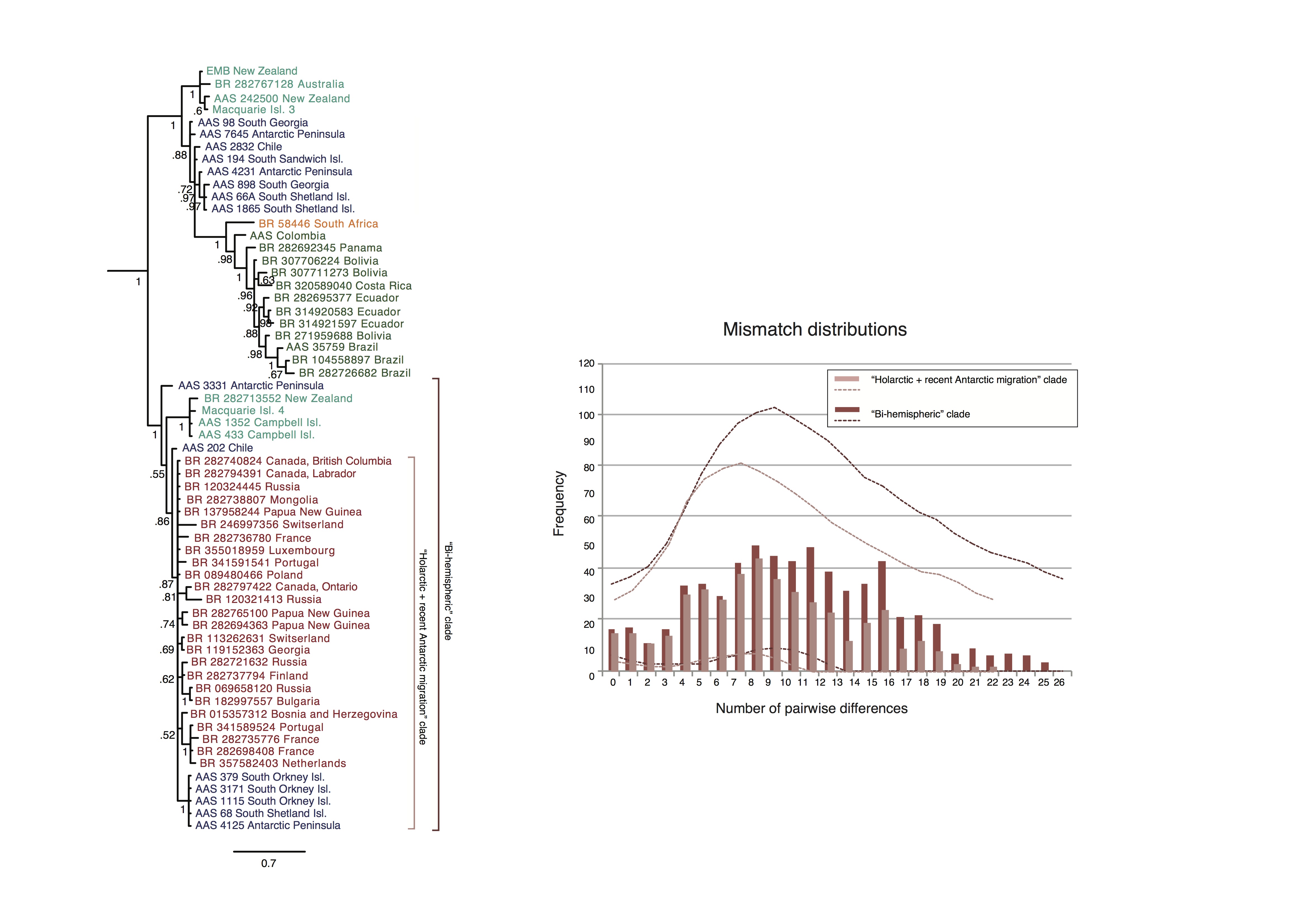


**Fig. S5** (related to Table 1 and Fig. 2). Observed mismatch distributions (bars) of sub-clades within *Polytrichum juniperinum* compared to the lower and upper bound of the expected distribution (dashed lines).

**References**

1 Drummond, A. J., Suchard, M. A., Xie, D., Rambaut, A. 2012 Bayesian phylogenetics with BEAUti and the BEAST 1.7. *Molecular Biology and Evolution.* **29**, 1969-1973. (10.1093/molbev/mss075)

2 Rambaut, A., Suchard, M. A., Xie, D., Drummond, A. J. Tracer v1. 6. Available from <http://beast.bio.ed.ac.uk/Tracer> 2014.

3 Bell, N. E., Kariyawasam, I. U., Hedderson, T. A. J., Hyvonen, J. 2015 *Delongia* gen. nov., a new genus of Polytrichaceae (Bryophyta) with two disjunct species in East Africa and the Himalaya. *Taxon*. **64**, 893-910. (10.12705/645.2)

4 Newton, A., Wikström, N., Beil, N., Forrest, L., Ignatov, M. 2007 Dating the diversification of the pleurocarpous mosses. In *Pleurocarpous mosses. Systematics and evolution. The Systematic Association special volume series 71*. (eds. A. E. Newton, R. Tangney), pp. 337–366. Boca Raton: CRC Press.

5 Ritzkowski, S. Year Das geologische Alter der bernsteinführenden Sedimente in Sambia (Bezirk Kaliningrad), bei Bitterfeld (Sachsen-Anhalt) und bei Helmstedt (SE-Niedersachsen). B. Kosmowska-Ceranowicz, H. Paner, editors. Investigations into amber: Proceedings of the international interdisciplinary symposium; Baltic amber and other fossil resins. 1999; Gdansk, Museum of the Earth, Polish Academy of Sciences. p. 33-40.

6 Konopka, A. S., Herendeen, P. S., Merrill, G. L. S., Crane, P. R. 1997 Sporophytes and gametophytes of Polytrichaceae from the Campanian (Late Cretaceous) of Georgia, USA. *International Journal of Plant Sciences*. **158**, 489-499.

7 Hyvonen, J., Koskinen, S., Merrill, G. L., Hedderson, T. A., Stenroos, S. 2004 Phylogeny of the Polytrichales (Bryophyta) based on simultaneous analysis of molecular and morphological data. *Molecular Phylogenetics and Evolution.* **31**, 915-928. (10.1016/j.ympev.2003.11.003)

8 Christopher, R. A. 1979 Normapolles and triporate pollen assemblages from the Raritan and Magothy Formations (Upper Cretaceous) of New Jersey. *Palynology*. **3**, 73-121.

9 Huddlestun, P. F., Hetrick, J. H. 1991 *The stratigraphic framework of the Fort Valley Plateau and the central Georgia Kaolin District*. Atlanta: Georgia Geological Society.

10 Gradstein, F. M., Ogg, G., Schmitz, M. 2012 *The Geologic Time Scale 2012*. Elsevier.

11 Hartmann, F. A., Wilson, R., Gradstein, S. R., Schneider, H., Heinrichs, J. 2006 Testing hypotheses on species delimitations and disjunctions in the liverwort *Bryopteris* (Jungermanniopsida: Lejeuneaceae). *International Journal of Plant Sciences*. **167**, 1205-1214.

12 Lang, A. S., Bocksberger, G., Stech, M. 2015 Phylogeny and species delimitations in European *Dicranum* (Dicranaceae, Bryophyta) inferred from nuclear and plastid DNA. *Molecular Phylogenetics and Evolution.* **92**, 217-225. (10.1016/j.ympev.2015.06.019)

13 Les, D. H., Crawford, D. J., Kimball, R. T., Moody, M. L., Landolt, E. 2003 Biogeography of discontinuously distributed hydrophytes: a molecular appraisal of intercontinental disjunctions. *International Journal of Plant Sciences*. **164**, 917-932.

14 Drummond, A. J., Rambaut, A. 2007 BEAST: Bayesian evolutionary analysis by sampling trees. *BMC Evolutionary Biology*. **7**, 214. (10.1186/1471-2148-7-214)
